# Is there equity of patient health outcomes across models of general practice in Aotearoa New Zealand? A national cross-sectional study

**DOI:** 10.1186/s12939-023-01893-8

**Published:** 2023-05-04

**Authors:** Nicolette Sheridan, Tom Love, Timothy Kenealy, The Primary Care Models Study Group, Nelson Aguirre-Duarte, Bruce Arroll, Carol Atmore, Jenny Carryer, Peter Crampton, Anthony Dowell, Tana Fishman, Robin Gauld, Matire Harwood, Karen Hoare, Gary Jackson, Rawiri McKree Jansen, Ngaire Kerse, Debra Lampshire, Lynn McBain, Jayden MacRae, Jane Mills, John Øvretveit, Teuila Percival, Roshan Perera, Martin Roland, Debbie Ryan, Jacqueline Schmidt-Busby, Tim Stokes, Maria Stubbe, Sarah Hewitt, Daniel Watt, Chris Peck

**Affiliations:** 1 Massey University, Auckland, Aotearoa New Zealand; 2 Sapere Research Group, Wellington, Aotearoa New Zealand; 3 University of Auckland, Auckland, Aotearoa New Zealand; 4 Comprehensive Care, Aotearoa Auckland, New Zealand; 5 WellSouth Primary Health Network, Dunedin, Aotearoa New Zealand; 6 University of Otago, Dunedin, Aotearoa New Zealand; 7 University of Otago, Wellington, Aotearoa New Zealand; 8 Independent Researcher, Auckland, Aotearoa New Zealand; 9 Te Whatu Ora - Health New Zealand Counties Manukau, Auckland, Aotearoa New Zealand; 10 Te Aka Whai Ora - Māori Health Authority, Auckland, Aotearoa New Zealand; 11 Datacraft Analytics, Auckland, Aotearoa New Zealand; 12 La Trobe University, Melbourne, Australia; 13 Karolinska Institutet, Stockholm, Sweden; 14 Health Hawkes Bay - Te Oranga o Te Matau-a-Māui, Hastings, Aotearoa New Zealand; 15 University of Cambridge, Cambridge, United Kingdom; 16 Pacific Perspectives, Wellington, Aotearoa New Zealand; 17 Te Whatu Ora - Health New Zealand, Wellington, Aotearoa New Zealand

**Keywords:** Models of care, Primary care, Patient health outcomes, Ambulatory sensitive hospitalisations, Polypharmacy, Immunisations, Emergency department attendance, Māori, Pacific, Deprivation

## Abstract

**Background:**

Primary care in Aotearoa New Zealand is largely delivered by general practices, heavily subsidised by government. Te Tiriti o Waitangi (1840) guarantees equal health outcomes for Māori and non-Māori, but differences are stark and longstanding. Seven models of primary care have evolved. We hypothesised that patient health outcomes would differ between models of care; and that Māori, Pacific peoples and those living in material deprivation would have poorer outcomes from primary care.

**Methods:**

We conducted a cross-sectional study of patient-level data from national datasets and practices, at 30 September 2018, using multilevel mixed effects regression analyses (patients clustered within practices). Primary outcomes, considered to be measures of unmet need for primary care, were polypharmacy (≥ 65 years), HbA1c testing in adults with diabetes, childhood immunisations (6 months), ambulatory sensitive hospitalisations (0–14, 45–64 years) and emergency department attendances.

Explanatory variables adjusted for patient and practice characteristics. Equity, by model of care, ethnicity and deprivation, was assumed if they showed no significant association with patient outcomes.

Patient characteristics included: age, ethnicity, deprivation, multi-morbidity, first specialist assessments and practice continuity. Practice characteristics included: size, funding and doctor continuity. Clinical input (consultations and time with nurses and doctors) was considered a measure of practice response*.*

**Results:**

The study included 924 general practices with 4,491,964 enrolled patients. Traditional practices enrolled 73% of the population, but, on average, the proportion of Māori, Pacific and people living with material deprivation was low in any one Traditional practice. Patients with high health needs disproportionately enrolled in Māori, Pacific and Trust/NGO practices.

There were multiple associations between models of care and patient health outcomes in fully adjusted regressions. No one model of care out-performed others across all outcomes. Patients with higher health need received more clinical input but this was insufficient to achieve equity in all outcomes. Being a Māori or Pacific patient, or living in material deprivation, across models of care, remained associated with poorer outcomes.

**Conclusions:**

Model-level associations with poor patient outcomes suggest inequity in measures that might be used to target investment in primary care.

**Supplementary Information:**

The online version contains supplementary material available at 10.1186/s12939-023-01893-8.

## Background

### Historical context

Fairness is a central value of Aotearoa^1^ New Zealand society [1, 2]; fairness is a matter of social justice [3]. However, health outcomes for Māori, the indigenous population, have been starkly worse than for non-Māori for more than a century [4]. Te Tiriti o Waitangi, an agreement between the British Crown and Māori signed in 1840, placed responsibility on the Crown “to protect actively Māori health and wellbeing through the provision of health services” [5]. Despite this, inequity remains embedded in health services, a legacy of the effects of colonisation and institutional racism [5]. Furthermore, persistent inequities for Pacific peoples and those living with material deprivation blight our health statistics [6].

Primary care in Aotearoa New Zealand operated on a fee-for-service basis from 1938 to 2001, part paid by government, and part paid by patients (a low proportion of whom were insured). Most care was provided by general practices, run as self-employed businesses with substantial government funding; this continues to be the basis of Traditional practices and the more recent development of Health Care Home (HCH) practices. From 1970 a practice nurse subsidy scheme supported general practitioners (GPs) to employ registered nurses to “provide a doctor’s assistant” [7]. By 1999 there was a nurse in 94% of practices [8] and a shift towards teamwork. Non-profit primary care organisations proliferated through the 1980s and 1990s – most notably Māori initiatives – in response to demands for affordable, culturally safe care [9]. General practice size increased through the 1990s, halving the number of solo-doctor practices from 32% in 1990 to 17% by 1999 [8], in response to government policy changes in contracting for health services [9].

A major change in health service delivery in 2001 came with a policy intent to shift towards primary health care [10], with its emphasis on addressing health inequalities [11] and attaining better health services for all [12]. The bulk of government funding shifted to a capitation formula, while retaining an option of patient part-payments. Capitation was adjusted primarily for the number, age and gender of patients enrolled in each practice, and whether practices contracted for additional payment together with a cap on patient co-payments in the Very Low Cost Access (VLCA) scheme [13]. Other funding adjustments were introduced over the years, for example, free consultations for children and limits to patient fees for prescription medicines. In this context several models of primary care delivery have evolved, mostly since 2001, providing the opportunity to compare models.

Along with these changes, other trends have shaped general practice. Private corporations have acquired existing practices as a business investment. Primary Health Organisations (PHOs), Government District Health Boards (DHBs) and community trusts have taken over some Traditional practices to ensure service to underserved geographic regions or communities. There has been an increase in the number of Māori health provider organisations and, more recently, Pacific health provider organisations, to address unmet health and social need in their communities. Our study was able to develop a typology of seven categories of primary care organisational models.

The Government has developed policy and long-term plans to build a workforce that reflects diverse communities [14]. There has been an increase in the number of health care assistants (HCA) supporting clinicians under delegated authority. Other roles include a registered profession of Fully Authorised Vaccinators and more recently, in response to the COVID-19 pandemic Vaccinating Health Workers, [15] and, as part of primary mental health and wellbeing initiatives, a registered profession of Health Improvement Practitioner [16]. The role of nurses in general practice has expanded, supported by government scholarships [17]. The number of nurse prescribers and nurse practitioners (NP) was starting to increase at the time of this study. The patient-centred medical home has been adapted to Aotearoa New Zealand as the “Health Care Home” [18], and has been actively promoted by some DHBs and PHOs [19].

The health system in Aotearoa New Zealand is in the midst of reform under the Pae Ora (Healthy Futures) Act 2022. A new Māori Health Authority (Te Aka Whai Ora) represents a new level of partnership that prioritises Māori health need, alongside that of the total population. The Act signals attention to locally focussed care (localities) but is otherwise silent on primary health and community care. Meanwhile, the Budget for 2022–23 [20] ignores long-term under investment in this area. Te Whatu Ora—Health New Zealand (a new entity to commission and deliver health services, including hospital and specialist health services) is responsible for the commissioning of primary health and community care. Overall, reform legislation, budgets and structures have downplayed the potential of the primary health and community care sector to improve health outcomes and equity [21]. Evidence is needed to support targeted investment in primary health and community care commissioning.

### This study

The New Zealand Ministry of Health and the Health Research Council of New Zealand sought research to guide investment in practice models that gave best patient health outcomes. The models they identified initially were Traditional, Corporate and Health Care Home. We defined these further and extended the classification to recognise a further four models; Māori practices, Pacific practices, and owned by a PHO/DHB or a Trust/Non-governmental organisation (NGO).

Studies of patient health outcomes in primary care can be grouped into those that evaluate a model of care, such as the patient centred medical home [22], those that consider specific features of primary care, such as continuity of care, access and the role of nurses [23–25], or features of individual practitioners [26]. Practice and practitioner processes may be considered together within a quality framework [27]. Many studies measure quality of care for a single condition, such as diabetes [28]. There is no single agreed set of outcomes [29]. Studies vary in the extent to which they adjust for patient need and few address issues of equity.

The aim of this study was to determine whether differences in patient health outcomes could be attributed to models of practice, after adjustment for other factors; whether these differences implied equity or inequity at the level of model of care; and whether we could explain the findings in terms that could indicate improvements to models of care. In regressions that adjust for ethnicity, material deprivation and other markers of patient need, an absence of significant association between a model of care and an outcome would imply equity at model-level. Significant associations with better outcomes would imply a model-level characteristic that might be applicable to other models.

Our priority was to consider the care of those less well-served by the current health system; this is disproportionately people who are Māori, Pacific, or living with material deprivation. The inquiry was at the level of general practice rather than individual practitioner. This paper reports on models of care and patient health outcome in the general population. Results from analyses on Māori-only and Pacific-only populations are reported elsewhere in the same collection of papers in this Journal.

## Methods

A cross sectional, observational study was conducted of all Aotearoa New Zealand general practices and enrolled patients as of 30 September 2018. The date was chosen because funding changes in December 2018 were likely to confound data interpretation. Further detail on methods can be found in Supplementary file 1. Qualitative data will be reported elsewhere.

### Data sources

Data came from national datasets, held by the Ministry of Health, and from practice information held by PHOs. Almost all general practices belong to a PHO, which at the time of the study contracted to a DHB to provide primary care services. DHBs were responsible for publicly funded health services in a geographical area/district; they ceased to exist as independent entities from 1 July 2022, replaced by the national entity Te- Whatu Ora – Health New Zealand. National datasets included PHO registers, inpatient, outpatient, laboratories, pharmaceutical dispensing, immunisations, the Virtual Diabetes Register (VDR), NZDep2018, the Index of Multiple Deprivation (IMD) and the Measuring Multimorbidity Index (M3), all available at patient level.

The VDR lists all individuals considered to have diabetes at 31 December 2018 based on linking six national administrative datasets [30]. The reported positive predictive value in 2014 was 82.3% and negative predictive value was 98.0%, higher in ethnic groups with a high prevalence of diabetes and in areas of highest material deprivation [31]. The NZDep2018 Index of Deprivation combines nine variables from the 2018 census which reflect eight dimensions of material deprivation, assigning a score to each resident in a small area generally including 100 to 200 people [32]. The IMD assigns a deprivation score to each geographic data zone; this score is attributed to individuals resident in that zone, averaging about 700 people per zone. The index is constructed from seven domains, which can be used independently: employment, income, crime, housing, education, health, and access. We used all domains except health [33]. The M3 index is a score assigned to each individual based on number and type of conditions they have, derived from hospital discharge coding [34]. Data from these sources were available for all practices and patients.

The national PHO register lists patients enrolled in each practice. A patient unique identifier, their National Health Index (NHI), is used throughout the health system. Processes linking patient-level data using an encrypted NHI are well-established.

Every practice in Aotearoa New Zealand uses an electronic medical record. All PHOs extract data from practices, although details vary between PHOs. Ten PHOs, with 292 practices, contributed patient-level data. From the appointment books calculated number and length of consultations and the profession of the clinician seen, representing face-to-face consultations, but not telephone, email or other contacts.

The practice data also allowed calculation of three items of preventive care: rates of cervical screening, cardiovascular risk assessment and HbA1c testing (the latter also drawing on data from the national laboratory dataset). Defining guideline-recommended eligible populations required data that were not available. For example, cardiovascular risk assessment is recommended for a population defined by age, gender, ethnicity, family history and additional risk factors. Therefore, while numerators are accurate, denominators included persons who were not eligible for each process so the calculated rates may be lower than a “true” measure. However, since the same method is applied to all practice models, relative differences between models are assumed to remain valid.

The workforce numbers and Full Time Equivalents come from a practice survey, specific to this study, sent to practices by all participating PHOs, so are self-reported. Not all practices received the survey. Date on GP FTE came from 370 practices, and RN FTE came from 367 practices but not all data were complete and comparable. The FTE calculations were based on data that covered 12% of patients.

### Defining practice models

#### Traditional practice

Typically centred upon the general practitioner, with mainly nursing support, operating as a small business, and owned by one or more doctors. These ranged from small to large organisations and served both high need and lower need populations. This is the longest-standing model and constitutes the majority of practices. Individual practices have a high degree of autonomy over service delivery.

#### Corporate practice

A group of practices owned and run as a for-profit business entity. Some delivered high volumes of care, with low costs for patients and often without the need for an appointment. Corporate practices had a relatively high degree of standardisation in business and clinical processes and information technology across different sites. Most corporate practices were Traditional practices before being bought by a corporate entity.

#### Health Care Home (HCH)

An adaptation of the Patient Centred Medical Home; the New Zealand HCH Collaborative maturity matrix focuses on business efficiency and sustainability [18]. It is a relatively new concept in Aotearoa New Zealand, with the first practice formally enrolling in the programme in 2011. Only 14 had been fully certificated as mature HCHs by 30 September 2018 (A Maxwell, personal communication 2018). At the time of this study those not certificated were at different stages of meeting the maturity matrix criteria. Most had been Traditional practices prior to embarking on the HCH programme.

#### PHO/DHB practices

Practices owned by a PHO or a DHB. This was a small group that had mostly been taken over by a PHO or DHB to continue to provide primary care services in a specific location, often an underserved and/or rural area.

#### Trust/NGO practices

One or more practices owned by an entity that was a not-for-profit Trust or NGO. They had a stated purpose, identifying a health or social goal. Many were in small communities or served populations with high need. Some provided, for example, salary and premises to attract and retain staff.

#### Māori practices

Practices owned and governed by Māori organisations, serving Māori and non-Māori patients. They were identified through lists from the Ministry of Health and DHBs together with web searches, direct contact with practices or were known to investigators. There may be a small number of practices we did not identify as Māori practices.

#### Pacific practices

Practices owned and governed by Pacific organisations, serving mostly Pacific and some non-Pacific patients. They were identified through lists from the Ministry of Health and DHBs together with web searches, direct contact with practices or were known to investigators. There may be a small number of practices we did not identify as Pacific practices.

Traditional, Corporate, PHO/DHB or Trust/NGO were considered to be ownership types. We assigned every practice to one of these ownership types although some practices were difficult to categorise. HCH, Māori and Pacific practices could overlap with ownership types, and HCH could overlap with Māori and Pacific practices.

### Patient health outcomes

Outcome measures were selected from existing performance indicators within collections of the New Zealand Health Quality and Safety Commission or the New Zealand Health Quality Measures collections, with already specified technical measurement standards [35]. Measures were known to show significant inequities between groups by health need, material deprivation or ethnicity but none had previously been examined for variation by primary care model of care. Supplementary file 4 shows potential practice outcome measures and sources of data, showing a large initial list which was reduced by consensus amongst the investigators. The six study outcomes used were as follows.

- *Polypharmacy:* Patients over 64 years old taking 5 or more long term medications over two consecutive quarters [36].
- *HbA1c Testing:* Patients on the national VDR with one or more HbA1c test in the previous year.
- *6 Month Immunisation:* Children who had received, by age 6 months, all the scheduled childhood immunisations up to and including those due at 5 months. The calculation includes only children who were 6 months old at some point during the analysis period. The Ministry of Health definition of on-time immunisation allows for a window of 1 month after the due date [37].
- *Child ASH Admissions:* The number of ambulatory sensitive hospital admissions for children who were under 15 years of age at the end of the analysis period [38, 39].
- *Adult ASH Admissions:* The number of ambulatory sensitive hospital admissions for adults who were between 45 and 64 years of age at the end of the analysis period [39].
- *ED Attendances:* The number of attendances at an ED for each patient over the analysis period.

The analysis period was the year 1 October 2017 to 30 September 2018. All measures used the national data sets. No adjustment was made for reduced data from those who died during the year of observation. Three outcomes were process measures: polypharmacy, HbA1c testing and childhood immunisations. Three were measures of intermediate outcomes: child and adult ASH and ED attendances. Better outcomes were assumed to be lower polypharmacy, ASH and ED attendances, and higher HbA1c testing and childhood immunisations.

### Explanatory variables

Supplementary file 3 shows lists of potential indicators of practice and patient characteristics. These lists were reduced by investigator consensus on data availability and priori*ty.*

#### Patient characteristics

Patients were assigned to the practice in which they were registered in the national PHO database at 30 September 2018. Age, gender and ethnicity were available at that date. Living in a deprivation area quintile 5 (most deprived quintile, Q5), Index of Multiple Deprivation (IMD) score of the area the patient lives in, distance to the nearest ED, the M3 score and being on the VDR all used 2018 data. Having gout was determined from dispensing data back to 2001 and hospital discharge data back to 1988 [40]. Having gout and diabetes are both associated with other long term conditions [41]. Being dispensed a selective serotonin reuptake inhibitor (SSRI, usually for depression), dispensed tramadol (for moderate to severe pain), dispensed an antibiotic, patient changing enrolled practice (a measure of practice continuity), and number of first medical specialist assessment (FSA) attended and not attended were all measured in the year 1 October 2017 to 30 September 2018.

#### Practice characteristics

VLCA practices agree to receive increased capitation funding while limiting their fees to patients. Practice uptake of this contract is voluntary subject to having an enrolled population of ≥ 50% Māori, Pacific or people living in Quintile 5 areas. Practices were designated as either urban or rural based on the rural status of a majority of their enrolled patients. The percentage of patient consultations, in the previous year, with the same GP, was used as a measure of personal continuity.

#### Primary care clinical input

Face-to-face appointments, recorded in the practice appointment record, were attributed to a RN, NP, GP or Other. Other included health care assistants, dieticians, physiotherapists, Quit smoking providers and unidentified persons. Total Consultations refers to the number of consultations recorded in the appointment book with a GP or NP in the previous year. There were low numbers of NPs and NP consultations so we made a decision to combine with GP. Low numbers of NPs would have been difficult to interpret. By combining with GP we were able to see more clearly the effect on patient outcomes of an independent consultation which a GP and NP did routinely. Time spent with each patient was cumulated to a proportion of Full Time Equivalent (FTE) per 1000 enrolled patients, separately for GPs, NPs and RNs, where that information was available.

#### Uncertainty in classification

Although explanatory variables were divided into three categories – patient characteristics, practice characteristics and clinical input, it is clear that some factors fall into more than one category. For example, attending a VLCA practice might be patient choice due to lower fees or a practice financial decision in response to local poverty. Having a First Specialist Assessment reflects both patient need and a referral from primary care in response to that need. Did Not Attend a FSA might indicate a patient barrier to access. Total Consultations, GP and registered nurse (RN) FTE can also be seen as markers of both patient need and system response. Patients changing practice within a year might reflect dissatisfaction with a practice but are more likely to reflect changing patient circumstances such as change of address, itself a correlate of poverty. However, changing patient-level or practice-level classifications will not alter regression findings in a model-level analysis.

### Regression analyses

Multilevel mixed effects regression analyses used patient-level data adjusted for clustering at practice level. All analyses were conducted in R statistical software [42, 43]. Variables that do not appear in the final regressions were not statistically significant in development models. The comparators used in the regressions vary between practice models. Ownership categories Corporate, PHO/DHB and Trust/NGO were compared to Traditional. HCH, Māori practices and Pacific practices were compared with not-HCH, not-Māori practices and not-Pacific practices, respectively. Variance was partitioned at the level of patient and practice, but not at level of model of care because a given practice might be classified to more than one model.

Practice model, being Māori, being Pacific and living in deprivation were entered as independent explanatory variables, and interpreted to imply inequity if there was a significant association between any of these variables and patient health outcome. Statistical significance is cited at *p* ≤ 0.05, with no adjustment for repeated modelling and multiple outcomes. Estimations for child ASH, adult ASH and ED attendances were run on 50%, 25% and 12.5% samples of the data respectively, to avoid excessively long computations with the negative binomial regressions used for this count data.

## Results

At 30 September 2018 there were 988 practices in the national PHO dataset. A small number of practices were combined where direct enquiry confirmed a single practice had registered its patients under individual doctors, or where parent and satellite clinics operated a single practice. Others were excluded as not relevant to our research question (rest-home services, youth-only services, student services) or practices that opened or merged, closed or changed PHO during the analysis period, which impacted on data collection. This left 924 practices, with 4,491,964 enrolled patients, as the subject of this study. We classified each practice to one ownership category (top row in Table 1), and, where relevant, as also Māori practices or Pacific practices or Health Care Homes. Most Māori practices (59/65) and Pacific practices (11/15) were owned by a Trust or NGO. Not able to be shown in the table, is that 10 practices were HCHs and Māori and Trust/NGO; one practice was HCH, Māori and Corporate; and one practice was HCH Pacific and Trust/NGO.

**Table 1:** Practice models showing overlapping categories, at 30 September 2018

	**Traditional** (*n* = 695)	**Corporate** (*n* = 103)	**PHO/DHB** (*n* = 27)	**Trust/NGO** (*n* = 99)
**Māori** (*n* = 65)	3	3	0	59
**Pacific** (*n* = 15)	4	0	0	11
**HCH** (*n* = 127)	90	14	7	16
**No overlapping model**	598	86	20	13

VLCA contracts (with lower patient co-payments), were held by 94% of Māori practices, 87% of Pacific practices, 78% of Trust/NGO, 56% of PHO/DHB, 35% of Corporate, 23% of HCH and 21% of Traditional practices.

Overall, 17% of practices were considered rural; the proportion was higher for Māori practices (34%), PHO/DHB practices (37%), Trust/Other practices (37%) and HCH (21%); models with lower percentages were Traditional (14%) and Corporate practice (11%); no Pacific practices were rural.

There were marked differences between the populations enrolled at the practice models, shown in Table 2. The majority of patients (73%) were enrolled in Traditional practices, with smaller proportions of Māori (59%), Pacific (49%) and people living in quintile 5 deprivation areas, of any ethnicity (56%). Conversely, Corporate practices, with 17% of total patient numbers, served 33% of Pacific people and 23% of the quintile 5 population. Māori practices and Pacific practices enrolled 19% and 10%, respectively, of all Māori and Pacific patients. While Trust/NGO practices enrolled 8% of the population, the proportions of enrolled patients who were Māori, Pacific or people living in deprivation quintile 5 were 19%, 17% and 17%, respectively.

**Table 2:** Patients enrolled: practice model by ethnicity and deprivation, at 30 September 2018

**Practice model**	**Enrolled patient populations**			
	**All patients** (*n* = 4,491,965)	**Māori** (*n* = 660,752)	**Pacific** (*n* = 312,670)	**Quintile 5** (*n* = 872,028)
**Corporate**	745,512 (17%)	119,585 (18%)	102,710 (33%)	202,297 (23%)
**Traditional**	3,261,719 (73%)	390,895 (59%)	152,756 (49%)	491,890 (56%)
**PHO/DHB**	142,507 (3%)	22,600 (3%)	4,548 (1%)	29,139 (3%)
**Trust/NGO**	342,226 (8%)	127,672 (19%)	52,656 (17%)	148,702 (17%)
**Māori**	241,503 (5%)	124,854 (19%)	21,544 (7%)	120,449 (14%)
**Pacific**	48,233 (1%)	4,816 (1%)	29,715 (10%)	26,026 (3%)
**HCH**	909,690 (20%)	132,448 (20%)	52,253 (17%)	165,623 (19%)

Numbers do not add to 100% due to overlapping practice model categories

Three measures of preventive care were available, shown in Table 3. For cervical screening the denominator was all women age 18 and over; for cardiovascular risk assessment, all adults age 18 and over; and for HbA1c testing, all people known to have diabetes. The relevant clinical guidelines recommend these preventive care activities for more narrowly-defined target populations but the data were not available to specify the denominators more precisely. The rates given are therefore an underestimate.

**Table 3:** Preventive care undertaken in the previous year, for 292 practices, by practice model

**Practice model**	**Cervical screening per 1000 women**	**Cardiovascular risk assessment per 1000 adults**	**HbA1c testing per 1000 adults with diabetes**
**Traditional**	322	212	1934
**Corporate**	469	193	1855
**PHO/DHB**	330	302	1824
**Trust/NGO**	310	233	1693
**Māori**	394	238	1630
**Pacific**	332	319	1884
**HCH**	243	150	1784

Cervical screening and cardiovascular risk assessment from PHO data, HbA1c from national laboratory dataset

Variation across the practice models was about two-fold for cervical screening and cardiovascular risk assessment, and 19% for HbA1c testing. Corporate practices had the highest rate for cervical screening but were near the low end for cardiovascular risk assessment. Traditional had the highest rate for HbA1c testing. HCH practices had the lowest rates of cervical screening and cardiovascular risk assessment. Māori practices were intermediate for cervical screening and cardiovascular risk assessment but lowest for HbA1c testing. Pacific practices had the highest rate for cardiovascular risk assessment and were near the high end of HbA1c testing.

Further differences between models, in terms of profile of enrolled patients, are shown in Table 4. Trust/NGO, Māori and Pacific practice models, compared with other models, show: smaller practice size; higher levels of deprivation quintile 5, IMD, M3, percentage Māori or Pacific patients; higher levels of nurse FTE and ratio of nurses to doctors (considered as a response to health service need); and lower levels of practice continuity.

**Table 4:** Practice models showing differences between populations enrolled, at 30 September 2018. Variables are considered to reflect aspects of patient need

	**All practices**	**Traditional**	**Corporate**	**PHO/DHB**	**Trust/NGO**	**Māori**	**Pacific**	**HCH**
**N enrolled** ^a^	3622 2074–6189	3597 2040–5971	5527 3133–9858	3642 1990–6865	2712 1528–4985	2954 1528–4975	2356 1722–5141	5750 3822–9502
**Age ≤ 14** ^b^	19% 17–22	19% 16–22	21% 17–25	21% 17–25	23% 20–26	25% 22–27	25% 23–28	20% 18–22
**Age ≥ 65** ^b^	16% 12–21	19% 16–22	21% 17–25	21% 17–25	23% 20–26	11% 8–15	9% 6–12	16% 12–20
**Māori** ^b^	10% 6–17	9% 5–14	11% 7–18	11% 8–23	34% 10–71	62% 35–77	8% 6–13	10% 6–18
**Pacific** ^b^	2% 1–5	2% 1–4	3% 1–10	2% 1–3	3% 1–11	4% 2–9	68% 32–84	2% 1–5
**Quintile 5** ^b^	12% 4–29	11% 4–21	17% 7–32	13% 3–35	47% 23–65	58% 38–72	46% 36–66	11% 5–23
**IMD** ^a^	2882 2191–3775	2707 2095–3473	3205 2406–3945	2902 2340–3972	4331 3445–4901	4568 3987–5030	4623 3735–4906	2798 1917–3569
**With diabetes** ^b^	5% 4–7	5% 4–6	5% 4–7	5% 4–6	7% 6–8	8% 7–9	11% 7–13	5% 4–6
**Dispensed SSRI in last year** ^b^	7% 5–8	7% 6–9	7% 4–8	7% 5–8	5% 3–7	4% 3–7	3% 1–4	8% 7–9
**Dispensed antibiotic in last year** ^b^	40% 35–44	40% 35–44	40% 37–45	38% 33–41	39% 32–44	42% 37–45	47% 44–50	38% 34–40
**M3 **^**a**^	0.14 0.11–0.16	0.13 0.11–0.16	0.13 0.11–0.15	0.13 0.10–0.16	0.18 0.14–0.19	0.18 0.16–0.21	0.16 0.13–0.19	0.14 0.12–0.17
**Non-continuity of practice** ^b^	21% 17–27	20% 16–26	23% 18–31	22% 18–26	25% 19–32	27% 21–33	33% 24–42	21% 17–27
**Distance to nearest ED** ^c^	7.4	7.3	6.7	12.7	9.4	8.7	5.4	8.1
**FTE nurse: 1000 enrolled patients **^a^	0.54 0.38–0.72	0.51 0.37–0.68	0.58 0.38–0.71	0.64 0.47–0.77	0.90 0.67–1.01	0.79 0.68–0.97	0.87 0.67–0.96	0.59 0.50–0.80
**FTE GP: 1000 enrolled patients **^a^	0.63 0.51–0.79	0.63 0.51–0.80	0.60 0.51–0.72	0.63 0.51–0.65	0.69 0.51–1.00	0.68 0.55–0.90	0.67 0.57–1.01	0.60 0.52–0.77

^a^ Number median / 25^th^ – 75^th^ centile

^b^ Percentage median / 25^th^ – 75^th^ centile

^c^ Kilometres, median / 25^th^ – 75^th^ centile

The distance to nearest ED was largest in PHO/DHB practices, which fits with these typically being in small and/or rural areas. Pacific practices showed the lowest distance to ED, which fits with Pacific peoples being largely urban.

### Statistical models

The final statistical models for each of the six primary outcomes are summarised and compared in Table 5. Age, numerical *p*-values and interactions are omitted from the summary, but the full models are available in Supplementary file 2, Table 1.

**Table 5:** Summary of final models for patient health outcomes across 924 practices. Age effects and interactions omitted – full regression outputs in Supplementary file 2

**Variable** **(reference values)**	**Polypharmacy** **Age 65 + ** *N* = 399,227 *R*^2^ = 0.364	**HbA1c for those with diabetes** *N* = 133,985 *R*^2^ = 0.1366	**6 month childhood immunisations** *N* = 26,859 *R*^2^ = 0.0795	**Child ASH admissions** *N* = 511,845 R^2^ not applicable	**Adult ASH admissions** *N* = 655,088 R^2^ not applicable	**ED attendances** *N* = 2,500,000 R^2^ not applicable
**Overall average**	38.2%	86.9%	75.6%	31 per 1000 children	38 per 1000 adults	254 per 1000 patients
***Practice models***						
**Corporate** (Traditional)	37.5% (38.3%)	86.3% (87.0%)	74.3% (75.7%)	-9.3%	20.9% ***	1.4%
**PHO/DHB** (Traditional)	35.5% (38.3%)	86.5% (86.9%)	74.9% (75.6%)	-14.5%	10.0%	10.3%
**Trust/NGO** (Traditional)	38.1% (38.2%)	88.4% (86.7%)	79.3% (75.2%)	38.3% **	31.5% ***	15.4% **
**HCH Practice** (All others)	38.7% (38.1%)	86.2% (87.1%)	78.5% (74.8%) ***	5.6%	-5.4%	-11.2% ***
**Māori Practice** (All others)	34.7% (38.0%) *	82.9% (87.0%) **	61.8% (76.4%) ***	-0.4%	5.9%	9.6%
**Pacific Practice** (All others)	36.9% (38.2%)	83.6% (87.0%)	66.5% (75.7%) *	-8.5%	-12.1%	-15.1% *
***Patient characteristics***						
**Male** (Female)		87.5% (86.2%) ***			30.2% ***	
**Māori** (Not Māori)	37.8% (38.2%)	85.5% (87.1%) ***	68.4% (77.0%) ***	28.1% ***	27.4% ***	20.8% ***
**Pacific** (Not Pacific)	34.2% (38.3%) ***	85.5% (87.1%) ***	76.1% (75.6) *	40.2% ***	28.0% ***	19.5% ***
**Quintile 5** (Not Q5)		86.3% (87.1%) ***				
**IMD** (25^th^, 50^th^, 75^th^ centiles) (ASH&ED ref: average IMD)	36.0% *** 38.5% 41.0%		78.1% *** 76.1% 73.9%	-11.2% *** 0.5% 13.1%	-10.5% *** 0.5% 11.4%	-8.2% *** 0.0% 8.9%
**Diabetes** (No diabetes)	67.9% (32.9) ***				20.4% ***	
**Gout** (No gout)	65.4% (35.7%) ***	89.0% (86.5%) ***			9.6% ***	
**HbA1c** (No HbA1c)	40.8% (34.3%) ***					
**SSRI** (No SSRI)	59.1% (36.3%) ***					36.5% ***
**Antibiotic** (No antibiotic)	41.9% (35.2%) ***	88.0% (85.6%) ***		145.9% ***	130.2% ***	86.2% ***
**Tramadol** (No tramadol)					58.2% ***	173.9% ***
**M3** (25^th^, 50^th^, 75^th^ centiles) (ASH&ED ref: M3 = 0)	34.9% *** 38.0% 44.1%	88.3% *** 87.6% 86.3%		19.3% *** 64.8% 256.6%	20.4% *** 49.2% 118.6%	13.8% *** 32.8% 77.5%
**Continuity of practice** (No continuity)				-24.3% ***	-18.9% ***	-20.2% ***
**Distance to Nearest ED** (1, 20, 100 km) (ASH&ED ref: average distance)			76.8% *** 74.9% 66.0%		3.2% -1.3% -18.1%	6.1% *** -3.0% -33.4%
**First Specialist Assessment** (FSA 1, 2, 3) (ASH&ED ref: FSA = 0)	39.8% *** 42.1% 44.3%	87.8% *** 88.9% 90.0%		45.9% *** 112.9% 210.7%	45.1% *** 110.5% 205.5%	46.1% *** 113.6% 212.2%
**First Specialist Assessment** ** Did Not Attend** (FSA DNA 1, 2, 3) (ASH&ED ref: FSA DNA = 0)			67.9% *** 58.9% 49.2%	15.5% ** 33.4% 54.2%	50.9% *** 127.6% 243.3%	50.7% *** 127.2% 242.5%
***Practice characteristics***						
**VLCA** (not VLCA)		86.2% (87.3%) *	74.1% (76.3%) *			
**Urban** (Rural)	38.1% (38.9%)	86.7% (87.8%) *	75.9% (73.6%)	-3.4%	-0.3%	
**Continuity of GP** (25^th^, 50^th^, 75^th^ centiles) (ASH&ED ref: Continuity = 0)				-6.6% *** -8.6% -12.7%		
***Primary care clinician input***						
**GP + NP consultations** (Consultations 1, 2, 3) (ASH&ED ref: Consultations = 0)	25.4% *** 28.3% 34.6%	81.8% *** 83.1% 85.3%	73.8% *** 74.3% 75.2%	8.7% *** 18.2% 39.8%	8.1% *** 16.9% 36.7%	7.5% *** 15.5% 33.3%
**RN hours** (Hours 1, 2, 4) (ASH&ED ref: average hours)		86.0% *** 86.2% 86.6%	74.1% *** 74.3% 75.1%			
**GP hours** (Hours 1, 2, 4) (ASH&ED ref: average hours)			74.8% *** 75.3% 75.4%	0.4% 0.0% 0.0%		

• Polypharmacy, HbA1c testing and immunisation results are logistic regressions

◦ For binary variables, results are % of patients with that outcome, i.e. if variable = 1 (the result if variable = 0 is given in brackets)

◦ For continuous variables, results are % of patients with that outcome, at specified value of variable, e.g. 25^th^, 50^th^, 75^th^ centile, or 1, 2, 4 h

• ASH and ED results are negative binomial regressions

◦ For binary variables, results are % change from the reference value i.e. result if variable = 1 compared to value if variable = 0

◦ For continuous variables, results are % change, at stated values, relative to the stated reference value; e.g. at GP hours 1, 2 or 4 compared to average hours

• Traditional practice is used as a reference for Corporate, PHO/Trust and Trust/NGO practice models

◦ The value for Traditional practice is given in brackets. This value varies slightly as the R margin command does not implement MEM (Marginal Effect at the Mean). We coded our own version of MEM but it is an approximation and there is some variability

^*^p < 0.05, **p < 0.01, ***p < 0.001

Across the six outcome regressions, the bulk of variance was at patient level. The proportion of variance at practice level was 4% for polypharmacy, 7% for HbA1c testing, 10% for childhood immunisations, 6% for child ASH, 4% for adult ASH, and 17% for ED attendance.

### Practice models

The analysis found the following statistically significant associations between practice models and the six patient health outcomes, after adjusting for patient characteristics, practice characteristics and primary care clinical input. Percentages are relative differences from the comparator for each outcome.

- *Traditional:* Reference category.
- *Corporate:* Adult ASH rate 21% higher than Traditional.
- *PHO/DHB:* No statistically significant associations.
- *Trust/NGO:* Child ASH rate 38% higher, adult ASH rate 31% higher, ED attendances 15% higher than Traditional.
- *Māori practices:* Polypharmacy 9% lower, HbA1c testing 5% lower, childhood immunisations 18% lower than other practices.
- *Pacific practices:* Immunisation rate 12% lower, ED attendances 15% lower than other practices.
- *HCH:* Immunisation rate 4% higher, ED attendances 11% lower than other practices.

### Māori, Pacific, deprivation

Overall, Māori patients showed poorer outcomes on 5 of 6 measures, with no difference for polypharmacy. Pacific patients showed poorer outcomes on 5 of 6 measures, with an immunisation rate above the population average. Deprivation, measured by NZDep2018 quintile 5 or IMD scores, were significantly associated with all 6 outcomes. The effect of ethnicity remained after adjustment for material deprivation and vice-versa, and remained after adjustment for multiple patient and health service characteristics including practice model of care.

### Summary of significant associations with remaining explanatory variable

- Age was strongly associated with all outcomes (except childhood immunisations, which were assessed at a specific age), see full regression outputs in Supplementary file 2, Table 1.
- Male gender was associated with an increase in HbA1c testing and adult ASH.
- Deprivation (Quintile 5 rather than quintiles 1 to 4, or an increasing IMD score) was associated with increased polypharmacy, ASH, ED attendances, and a decrease in childhood immunisations. IMD at the 75^th^ centile was associated with 13.1% more child ASH than at the average IMD.
- Rural practice was associated with a small increase in HbA1c testing.
- VLCA practice was associated with a decrease in HbA1c testing and childhood immunisations.
- Having diabetes was associated with polypharmacy, and 20.4% more adult ASH than patients without diabetes.
- Having gout was associated with an increase in polypharmacy, HbA1c testing and adult ASH.
- SSRI dispensing was associated with an increase in polypharmacy and a 36.5% increase in ED attendances.
- Antibiotic dispensing was associated with an increase in polypharmacy and HbA1c testing; and increases in child ASH, adult ASH and ED attendances of 145.9%, 130.2% and 86.2%, respectively.
- Tramadol dispensing was associated with an increase of 58.2% in adult ASH and 173.9% in ED attendances.
- As M3 increased, polypharmacy increased and Hba1c testing decreased. M3 at the 75th centile (for patients with a non-zero score) was associated with 256.6% more child ASH, 118.6% more adult ASH and 77.5% more ED attendances (compared to average IMD for patients with a non-zero score).
- Continuity of practice was associated with a decrease in child ASH of 24.3%, adult ASH of 18.9% and ED attendances of 20.2%.
- As the distance increased from a patient’s home address to the nearest ED, there was a decrease in childhood immunisations, adult ASH and ED attendances.
- Continuity of GP, at the 75^th^ centile, was associated with a decrease in child ASH of 12.% compared to a child with no continuity.
- More FSA were associated with an increase in polypharmacy and HbA1c testing. Compared with no FSA, patients 3 FSA were associated with increases in child ASH, adult ASH and ED attendances of 210.7%, 205.5% and 212.2%, respectively.
- More FSA Did Not Attend: a count of 3 FSA DNA was associated with a 49.2% immunisation (noting the overall average was 75.6%). A count of 3 FSA was also associated with increases in child ASH, adult ASH and ED attendances of 54.2%, 243.3% and 242.5% respectively, suggesting a need for extra support to engage.

### Primary care clinician input

- More total consultations (GP and NP) were associated with increased polypharmacy, HbA1c testing and childhood immunisations. A count of 3 consultations, compared with none, was associated with increases child ASH, adult ASH and ED attendances of 39.8%, 36.7%, and 33.3%, respectively.
- More RN hours were associated with increased HbA1c testing and childhood immunisations. More GP hours were associated with increased childhood immunisations.

## Discussion

Patient characteristics explained the bulk of the variance in associations between practices and outcomes. Nevertheless, there remained sufficient variance at the level of practice to be considered a target of policy, interventions and ongoing data monitoring. The largest practice-level variance was 17% for ED attendance. The highest rate for ED attendance was in Trust/NGO practices, and the lowest in HCH and Pacific practices. The regressions further allowed us to assess the associations of seven practice models of care, and being Māori, being Pacific, or living in material deprivation, after adjusting for patient and practice characteristics and primary care clinical input. There were 36 associations between model of care and patient outcomes (six outcome regressions for seven models less a reference model). Twenty-four associations were statistically non-significant, indicating no difference in outcome attributable to model of care after adjustment for patient and practice characteristics.

At the level of model of care there were seven associations with worse patient outcomes and four associations with better patient outcomes. While some findings are small in absolute terms, it should be noted that, if these cross-sectional associations remain over time, the effect on patients is likely to be cumulative. Furthermore, there are likely to be similar small but cumulative differences accruing across multiple health outcomes we did not measure.

Broadly, models fall into two groups, based on patient profiles and outcomes in Tables 2, 3, 4 and 5; Traditional, Corporate, HCH and PHO/DHB; and Trust/Other, Māori and Pacific. This is consistent with the fact that most Corporate, HCH and PHO/DHB practices started life as Traditional practices. It is also consistent with the overlap of classifications Trust/Other, Māori and Pacific practices shown in Table 1.

### Traditional practices as a reference

In the regressions, Traditional practice was used as a reference group for the other ownership categories of Corporate, PHO/DHB and Trust/NGO. In 14 of 18 estimates, Traditional was not statistically different from these models. Because 73% of all patients were enrolled in Traditional practices, these had a dominant effect on the overall averages for each outcome. These averages can be directly compared with the estimates for other models of care and for explanatory variables. Table 3 shows Traditional practices to have the highest rate of HbA1c testing, while being mid-range for rates of cervical smears and cardiovascular risk assessment.

### Trust/NGO, Māori and Pacific practices

One or more of these practice models show worse patient outcomes for HbA1c testing, childhood immunisations, child ASH, adult ASH and ED attendances (Table 5). In each case worse outcome is also associated with increased GP and NP consultations combined, often also with increased GP and RN time. Together, this seems to show that practices increased clinical input in response to need, but the increase was not sufficient, or the most appropriate resource, to meet overwhelming patient need. That need, and related complexity, is shown in high levels of poverty (quintile 5, IMD) and comorbidity (M3, diabetes, and need for specific medications) (Table 4).

Most of these practices had low patient fees. Very Low Cost Access contracts were held by 94% of Māori practices, 87% of Pacific practices and 78% of Trust/NGO practices, compared with the national average of 30%.

Trust/NGO, Māori and Pacific models, compared to other models, had higher ratios of nurses to patients, GPs to patients, and nurses to GPs (Table 4). This suggests differences in function within the models, which are discussed further in the accompanying Nursing paper in this Journal.

### Māori practices

Many Māori Provider Organisations were established in the 1980s as predominantly nursing services that employed GPs. These services placed an emphasis on meeting the clinical and cultural needs of local communities. In this study 34% of Māori practices were considered rural which is twice that of the overall practice average (17%).

Patients enrolled in Māori practices showed a lower rate of polypharmacy in people over 64 than patients enrolled in other practice models. A beneficial effect of lower polypharmacy might be mediated by engagement in a culturally safe environment with gains, for example, in communication, health literary, and adherence to therapies and prescribed medications [44–46]. However, given the suggestion above that Māori practices are overwhelmed (together with Trust/NGO and Pacific practices), lower polypharmacy might indicate under-prescribing. Overwhelmed practices seem the likely explanation for lower rates of HbA1c testing and childhood immunisations.

### Pacific practices

ED admissions were lower in Pacific practices. These practices were all in urban areas. The enrolled population in a given practice often comprised (mostly) a single Pacific ethnic population using a shared first language. There is some evidence that language is a primary barrier to Pacific peoples engaging with primary care that, elsewhere, is largely conducted in English [47]. Pacific practices also have a high ratio of nurses to patients; all nurses speak English but many also speak one or more Pacific languages. Cultural concordance goes beyond language as noted above for Māori practices (this concept is discussed in another publication in this collection).

### Corporate practices

Adult ASH rates were higher in Corporate than Traditional practices. Corporate practices that serve large numbers of patients in urban, high need areas are typically accessible via longer opening hours and lower fees than are Traditional practices, suggesting they could be more readily available to provide care that reduces need for ED attendances or hospital admissions. It is possible that referral patterns from primary care to hospital varies between practice models, but we have no data to explore this. Furthermore, patients can attend ED and be admitted to hospital without going through primary care, but it is not known whether the rate of self-referral varies between models of care.

In terms of preventative care, Corporate practices achieved the highest rate for cervical screening, while being at the low end for cardiovascular risk assessment and mid-range for Hba1c testing.

There appear to be differences in prioritisation of preventive care between models of care, for reasons that are not clear.

Cardiovascular risk assessment attracted fee-for-service funding, as a national target, at the time of this study. HbA1c testing, as a component of a free diabetes annual review, had long been subject to a fee for service although this had ceased well before this study. Cervical screening has never been subject to national targets or a fee-for-service subsidy except in local initiatives for women considered high risk.

### Health care homes

The enrolled population profile for HCH practices showed lower patient need than for other practice models except Traditional. HCH practices were associated with a higher immunisation rate and fewer ED attendances but also the lowest rates of cervical screening and cardiovascular risk assessment. The HCH Collaborative emphasises systematic care processes and data recording [18]. It is uncertain how well developed the defined HCH features are in non-certificated practices that have started HCH implementation. At the time of this study only 14 of 127 HCH practices were certificated as mature examples of the HCH, with others newer to this style of practice. Early adopters of the HCH model were self-selected and were acknowledged to be high-functioning Traditional practices. As the model is spread further, the same level of performance may not be achieved in all practices. Nevertheless, all HCH practices have committed to uniform standards which may result in less variation between HCH practices than between practices in other models.

### Childhood immunisations

Immunisation at 6 months were lower in Māori practices and Pacific practices than for others. With the immunisation schedule requiring vaccines at 6 weeks, 3 months and 5 months, measuring immunisation rates at 6 months leaves a window of only one month in which to administer vaccines due at 5 months [37]. Residential mobility is one barrier to timely immunisation. The proportion of patients who changed practice in the previous year, in our data, was 21% overall but 27% in Māori practices and 33% in Pacific practices. While dissatisfied patients do change practice, it is perhaps more likely that these data reflect residential mobility, some of which will be associated with poverty [48]. Another barrier to immunisation is respiratory infection, a common reason to delay immunisation, and there is evidence of high rates of respiratory illness in Māori and Pacific children [49].

### Primary care clinical input

We considered the level of primary care clinical input (GP, NP, RN consultations and time) into the management of individual patients to be a marker of patient need. Secondary care clinical input via First Specialist Assessment is considered to be a measure of both patient need and primary care response by referral.

Primary care clinical input was associated, independent of practice models, with higher (worse) outcomes for polypharmacy, child ASH, adult ASH and ED attendances, and higher (better) outcomes for HbA1c testing and childhood immunisations. This suggests that, independent of model, primary care clinical input was more aligned to need for HbA1c and childhood immunisations but not for the other outcomes. This implies a need for more GPs, NPs, RNs and other health care workers, across models, to address hospital use in particular.

### HbA1c testing and child ASH

HbA1c testing is a direct measure of quality of care because it is a necessary step on the pathway to good glucose control. Associations with decreased HbA1c testing (Māori practice, Māori patients, Pacific patients, Quintile 5 deprivation, urban practice, M3) suggest this may be due to diabetes being lower priority in the presence of complexity and multimorbidity.

Respiratory illnesses, including pre-school asthma, contribute to the national statistics for child ASH [50]. Young children are vulnerable to rapid deterioration with respiratory illnesses and primary care clinicians appropriately send infants and children to hospital; some children go directly to hospital without attending primary care. The optimum rate of child ASH remains unknown [50]. However, statistically significant differences still indicate real practice-level and model-level variation.

### Differences in enrolled populations

Differences in patient health outcomes were associated more strongly with patient need than with practice model. Table 4 shows 15 measures, thought to indicate individual and population need; a markedly higher proportion of higher-risk patients were enrolled in Māori, Pacific and Trust/NGO practices than in other practice models. Taken together, these point to a raised workload necessary to respond to a concentration of complexity in Māori, Pacific and Trust/NGO practices, with direct implications for resourcing [51].

The majority (59%) of Māori patients and half (49%) of Pacific patients were enrolled in Traditional practices where they were typically a small percentage of enrolees in any one practice. Traditional practices face the challenge of how to specifically address cultural safety when caring for Māori, Pacific, and other ethnic groups, targeting their resources for equity for patients with high needs.

Trust/NGO, Māori and Pacific practices employed a higher ratio of nurses to patients, and nurses to doctors, than other models of care. They explicitly address both the health and social needs of patients and often work in a complex organisation. Most Māori and Pacific practices were constrained within the same funding streams as other practices, although some have received additional support through the Māori Provider Development Fund or the equivalent Pacific fund.

### Current resourcing options do not target the range of patient need

Equitable resource allocation is required to improve health outcomes for Māori, Pacific and people living with material deprivation. At the time of this study, funding and resources to support primary care were provided through a range of mechanisms that target financial support for health services to: individuals (Capitation and the High Use Card [52]; the individual and family (Community Services Card [53]); the whanau / household (Prescription subsidy scheme [54]); the practice at which the patient is enrolled (Very Low Cost Access (VLCA) [55]); specific services (DHB and PHO programmes e.g. palliative care); specific conditions (DHB and PHO disease management programmes) and on residential area material deprivation (Services to Improve Access). All these subsidies are paid to health care providers, and do help to reduce practice level cost to patients, but do little to address the multiple additional barriers to access [56], especially for patients with complex clinical or social circumstances. Some direct assistance to patients as provided through PHOs supporting locality-based resources – such as employing kaiawhina / community health workers who work across multiple practices, or channelled through other welfare support outside the health system, such as, disability allowance payments can cover some health care cost, administered through the Ministry of Social Development Work and Income services.

### Practice size

Practice size was not retained in any of the final models. It is possible that any effect of practice size was captured in factors that correlated with size such as rurality, workforce consultations and FTE and continuity of GP. The literature is mixed on the relationship of practice size to measures of process or outcomes. A report from the UK analysed data on practice size in relation to measures from the Quality and Outcomes Framework and rates of ASH [57]. They found that larger practices performed better, but with wide variation, and without being able to adjust for patient characteristics. A systematic review in 2013 included 13 cross-sectional studies. Five of 10 studies reported better scores on some processes of care in larger practices, while three studies of patient-reported outcomes found better access in smaller practices [58].

### Rurality

In many jurisdictions, rurality is a risk factor for poor health outcomes. This is likely to be due to associations of rurality and other known risk factors, including access to health services and socio-economic disadvantage compared to urban dwellers [59]. In the current study, rural patients are, by a small margin, more likely to have an HbA1c test, but otherwise rurality is not associated with the outcomes we measured, after adjustment for a wide range of patient characteristics. Rurality is associated with several measures that may influence health service access including greater distance to nearest ED, small practices, PHO/DHB practices and Trust/Other practices and Māori practices, and Māori but not Pacific ethnicity.

### Primary care data

Despite collecting the largest data set on primary care in Aotearoa New Zealand to date, we are aware of gaps in the data, such as for mental health care and the work of nurses and other health care workers, which remain largely invisible.

National datasets, which are largely collected from secondary care systems or administrative records such as prescriptions dispensed or tests undertaken, were largely clean and consistent, reflecting the substantial infrastructure behind them. However, significant effort was needed to clean and interpret national data from practice registers and records. Historically, the registers were assembled for capitation purposes, and did not always match the definition of a practice from other perspectives. For example, satellite clinics might be listed as separate entities or might be merged with the parent clinic. It is unclear whether the National Enrolment System, implemented since we extracted our data, will make this task easier in the future.

Extracting data from practices via PHOs presented significant challenges. Each PHO had a different approach to permitting data to be used, and the extent of data they collected routinely varied. 

There is a need to standardise data collection and analysis from all practices, using, for example, existing measures in the Atlas of Variation. There are important unresolved questions about who owns patient data and the extent to which analysis and reporting anonymises practitioners and practices preventing scrutiny that may be in the patient or public interest. Without data, inequity remains hidden [60] and the health and social systems cannot allocate resources to address equity.

We have identified markers of individual patient risk that could be used to help target resources. Those currently in use are age, gender, ethnicity and material deprivation as measured by Quintile 5. In addition, there are strong and consistent associations between patient health outcomes and M3 score, number of NP or GP consultations, FSA or not attending a FSA, change of practice enrolled in, and being dispensed an antibiotic or tramadol. Furthermore, material deprivation as measured by IMD was more consistently associated with patient health outcomes than was Quintile 5. We have identified models of care where most practices had high measures of accumulated patient health need (Māori, Pacific and Trust/NGO practices) compared to other models of care.

### 2022 Aotearoa New Zealand health reforms

The health system in Aotearoa New Zealand is being restructured under the Pae Ora (Healthy Futures) Act, which took effect on 1 July 2022. A central driver of reform is the need to address decades of disparities in health outcomes, especially for Māori. The 20 DHBs present when this study was conducted have been dis-established and health services now operate under a single national entity, Te Whatu Ora – Health New Zealand. Also newly established, Te Aka Whai Ora – Māori Health Authority, is an independent statutory authority to lead improvement in Māori health [61]. Te Aka Whai Ora is not a separate health system for Māori, but an entity to co-design and co-commission for the new health system.

Up to 80 community localities are planned across Aotearoa New Zealand to provide health service advice. The relationships between localities, PHOs and general practices, are in development. PHOs might be dis-established; many of their functions such as clinical improvement and service coordination will continue, albeit under another entity as yet not defined [62]. The primary care sector is unsettled. There are longstanding staffing shortages, COVID-19 has created additional demands for services and there is concern about a legacy of a range of unmet care needs, including preventive care.

### Study limitations

We acknowledge that measurement of associations cannot prove causality, that many factors affecting outcomes reside outside primary care and many remain unmeasured. These include the social determinants of health – racism, housing and others – which are only partially captured in the deprivation measures we have used. The purpose of regressions was to “remove” the effects of patient characteristics but this can never be done perfectly due to imprecise measurement of each concept for which the variables are a proxy, exacerbated when the differences between practice populations are large.

We have previously noted that, although explanatory variables were divided into three categories – patient characteristics, practice characteristics and clinical input, some variables likely contributed to more than one category. However, these categories are for clarity of discussion; category assignment does not affect the regressions.

Our explanations for differences between the practice models of care, as shown in the regression outputs, are based on available data and research team expertise. While the best outcome for individual patients remains unknown, we have assumed that, at a system level, a lower rate is better for polypharmacy, ASH and ED attendances, and a higher rate is better for HbA1c testing and childhood immunisations.

Trends over time for each outcome may have been more informative than a cross sectional analysis. However, such data were not available for all outcomes and variables, and would have been difficult or impossible to interpret due to patient turnover within practices (21% per year in our data) and major changes to practices with opening, closing or merging during the year studied (35 practices in our initial data) and periodic changes to practice funding policies.

We recognise that each practice had its own history of adapting to their enrolled patient population and region, and changes in policy and funding context. Grouping them together into “models” as we have done was a necessary simplification to address our research question.

## Conclusions

Practices with the highest proportions of enrolled patients with complex high health needs were consistently found in Māori, Pacific and Trust/NGO models of care. The Traditional model of care comprised the largest number of practices and, on average, the smallest number of patients with high need per practice. HCH practices had a similar patient profile to Traditional practices. Resources need to align more strongly with models of care and practices relative to the number of patients with high health needs.

Recent health system reforms (July 2022), including the establishment of Te Aka Whai Ora – the Māori Health Authority, provide a new opportunity to invest in primary care and prioritise Māori health outcomes.

We have identified markers of individual patient risk that could be used to help target resources, in addition to those currently used: deprivation as measured by IMD, M3 score, number of NP or GP consultations, number of FSA or not attending a FSA, change of practice enrolled in, and being dispensed an antibiotic or tramadol. We identified models of care where most practices had high measures of accumulated patient health need (Māori, Pacific and Trust/NGO practices).

An increase in the primary care workforce and standardised data collection in primary care is fundamental to improving patient health outcomes.

## Supplementary Information

**Additional file 1: Supplementary file 1.** Methods for quantitative data collection and analysis.

**Additional file 2: Supplementary file 2.** Full output of final regression models.

**Additional file 3: Supplementary file 3.** Potential indicators of practice and patient characteristics.

**Additional file 4: Supplementary file 4.** Potential practice outcome measures and sources of data.

## Data Availability

Data for this project were collected on condition of anonymity of patients, practices and PHOs, with an agreement to delete data once the purpose of the project was met. Data collected from practices and PHOs are not available. Data collated from national data sets is available in summary form on request to author TK (t.kenealy@auckland.ac.nz).

## Abbreviations

DHB: District Health Board; responsible for government-funded health services, including hospitals, in a geographical area; ceased to exist as independent entities from July 2022
ED: Emergency department of a secondary care hospital
FSA: First specialist assessment following referral to secondary care
FSA DNA: Did not attend first specialist assessment
FTE: Full time equivalent; refers to hours of work
GP: General practitioner (family physician)
HCH: Health Care Home
IMD: Index of multiple deprivation
M3: Multi-morbidity index
NGO: Non-governmental organisation
NP: Nurse Practitioner
PHO: Primary Health Organisation; almost all general practices belong to a PHO, which contracts to a DHB to provide primary care services
Q5: Quintile 5 (most socioeconomically deprived) on NZ Deprivation Index
RN: Registered nurse
SSRI: Selective serotonin re-uptake inhibitor; a widely used category of antidepressant
VDR: Virtual diabetes register; a national list of people considered to have diabetes
VLCA: Very low cost access; practice with higher subsidies and lower fees

## Footnotes

1 Aotearoa was the name given by Māori to this land. The other name, New Zealand, was given by a Dutch explorer hundreds of years later.
