# Supplementary material for: Is there equity of patient health outcomes across models of general practice in Aotearoa New Zealand? A national cross-sectional study
Source: Int J Equity Health. 2023 May 4;22:79. doi: 10.1186/s12939-023-01893-8 (PMC10157126; doi:10.1186/s12939-023-01893-8)
Supplement: Supplementary file 1 — Additional file 1: Supplementary file 1. Methods for quantitative data collection and analysis. [file 12939_2023_1893_MOESM1_ESM.docx]

**Supplementary file 1. Methods for quantitative data collection and analysis**

**Corresponding Authors**

Timothy Kenealy, [t.kenealy@auckland.ac.nz](mailto:t.kenealy@auckland.ac.nz)

Tom Love, [tlove@srgexpert.com](mailto:tlove@srgexpert.com)

Nicolette Sheridan, [n.sheridan@massey.ac.nz](mailto:n.sheridan@massey.ac.nz)

**Purpose of this Appendix**

This Appendix is intended to be a single source of information on methods used in a series of reports from the same dataset and reported in this collection within the International Journal for Equity in Health.

**Study design**

Cross sectional analysis at 30 September 2018. This includes calculating event rates over the previous year. This appendix records quantitative data collection and analysis. Twenty four accompanying case studies, which collected only qualitative data, are reported separately.

**Setting**

Conducted in Aotearoa (New Zealand) in 2019-2022. The health care system is described in the Background to the Primary Outcomes paper. The study date was chosen because funding changes in December 2018 were likely to confound data interpretation.

**Participant practices – national data**

All 988 practices in the national Primary Health Organisation (PHO) dataset at 30 September 2018 were potentially eligible, along with all patients enrolled in those practices. Practices were excluded if they had opened, closed or merged during the period of analysis (October 2017 to September 2018). Practices were also excluded if they were Youth One Stop Shops, student health services for a tertiary institution or were a rest home-only service, as data from these practices would not address our research question. When in doubt, practice status was confirmed by direct contact with practices. We considered excluding practices that changed PHOs during the period of analysis but decided against this on the basis that our investigation centred on whether practices were an important determinant of patient health outcomes, in which PHOs are one element of context. After exclusions 924 practices were included in the study. Data from national datasets were available for all 924 practices, regardless of the participation of the PHO they were contracted to.

**Participant practices – PHO data**

The CEO of every PHO was approached in person or by phone then followed procedures specific to each PHO. This typically involved submissions and personal presentations to clinical governance boards and repeated discussions with IT leads and practice liaison staff. Of 31 PHOs in existence at the time of this study, 21 agreed to some level of participation, and 10 PHOs with 292 practices eventually contributed patient-level data. Those that declined generally did not have the necessary data or the data management capacity available, although four also expressed concerns about how the data might be used.

Every practice in Aotearoa uses a computerised practice management system (PMS) but there were multiple software programmes in use. At the time of the study almost every practice belonged to a PHO, which contracted to a DHB to provide primary care services in a geographical area / district. DHBs were responsible for publicly funded regional health services; they ceased to exist as independent entities from 1 July 2022, being replaced by the national entity Te Whatu Ora – Health New Zealand

Every PHO collected data from their practices but they vary in what data was collected, how often, what data are held centrally by the PHO, and what agreement they have with practices with respect to making data available for research. Where practices within a PHO used different PMS software, PHOs could not always extract the same data from each PMS. In a small number of cases we worked directly with practices.

We requested from the PHOs, for the three years prior to 30 September 2018, extracts from the following data blocks: Encounters, Consults, Prescribing, Medication, Invoice, Appointment, Patient demographics, Referral, Recalls. No text records of clinical consultations were extracted.

We also requested practice descriptions from PHOs, which they provided from their own records or by sending a survey to their practices. Not all practices received the survey. By data cut-off at 3 April 2020 practice description data had been received from 768 practices in 21 PHOs and practice activity data from 452 practices in 13 PHOs. The requested data included numbers and full time equivalent (FTE) for each staff category. 370 practice provided data on GP FTE and 367 on RN FTE but not all data were complete and comparable. The FTE calculations were based on data that covered 12% of patients.

**Data sources - national datasets**

We collected three years of data (October 2015 to September 2018) for PHO registers, inpatient, outpatient, laboratories, immunisation, mental health and general medical subsidy data collections. We collected one year of data on dispensed pharmaceuticals (October 2017 to September 2018). The Ministry of Health also provided the New Zealand Deprivation Index (NZDep) 2018 [1], the Virtual Diabetes Register (VDR) 2018 [2], and the Measuring Multimorbidity (M3) score 2018 [3]. The University of Auckland provided the Index of Multiple Deprivation (IMD) score 2018 [4]. Data from these sources were available for all practices and all patients.

The national PHO register lists all patients enrolled in each practice at a given date. Included is the patient unique identifier, their National Health Index (NHI), used throughout the health system. Processes to link patient-level data using an encrypted NHI are well-established. At the time of this study PHO registers were constructed quarterly – collection is now in real-time.

National inpatient and outpatient data collections include all events from public hospitals, and some events from private hospitals. Each event is coded with International Classification of Disease (ICD) codes. Process codes include outpatient appointments attended or not, triage severity level in the Emergency Department, and length of hospital admission. Nearly all “medical” and “acute” hospital admissions are to public hospitals but about half of elective surgical procedures are carried out in private hospitals [www.nzpsha.org.nz; accessed 10 Dec 2020].

The national laboratory data collection records all publicly funded laboratory tests, including which test was done but not the result, and includes provider type and registration number. The national immunisation register records all publicly funded vaccinations delivered to children. The pharmaceuticals collection includes dispensed medication, dose and intended duration, and an identity-code for the prescriber. The general medical subsidy dataset can be used to investiture how often some groups of patients visit practices in which they are not enrolled. The national mental health collection records all publicly-funded contacts with community or secondary care mental health services but was not used.

The VDR lists all individuals considered highly likely to have diabetes on the basis of an algorithm run over national databases including hospital admissions, diabetes outpatient appointments, medication dispensing and laboratory testing [2, 5].

The IMD assigns a deprivation score to geographic data zones, where the average zone includes about 700 people. [4] This score is attributed to individuals resident in that zone. The IMD uses routinely collected data from government departments and census data. The index is comprised of 28 indicators in seven domains: employment, income, crime, housing, education, health, and access to services. The domains can be used separately or in combination. We used the sum of six domains, excluding health to avoid correlations with other measures [6] [4].

The M3 is a multi-morbidity score, applied to each individual within national databases. It is derived from the regression coefficients for the risk of 1-year mortality associated with 55 conditions identified from ICD codes in the national hospital inpatient data. No score is available for individuals who have never been to hospital. For our purposes these individuals are considered to have a score of 0 [3], which underestimates morbidity for conditions not associated with hospitalisation.

The NZDep index is a proxy score for deprivation using comparative socioeconomic positions of small areas and assigning them quintile numbers from 1 (least deprived) to 5 (most deprived) [7]. The index is based on 9 socioeconomic variables from the 2018 Census [1].

The Health Quality and Safety Commission conducts a national primary care Patient Experience Survey on an ongoing basis [8]. Data does not include a patient identifier but can be linked to practice with agreement from the practice or PHO or both. We obtained such permission from only a small number of practices and did not make use of this dataset.

**The research team**

The team comprised 28 named investigators from three universities in Aotearoa and two international universities, research companies, and hospital and health organisations.

The team were chosen for their academic and clinical knowledge of general practice and included academics with medical and nursing registration, with specialisations in primary care, public health, child and youth health, older persons health and mental health; and expertise in data management and analysis. Furthermore, the team included people in governance and advisory positions with practices, PHOs, DHBs, and the Ministry of Health. Māori and Pacific members of the team formed governance and provided guidance from within the team. A Steering Group comprised of health sector appointees by the Ministry of Health and the Health Research Council confirmed the aims and approach of the study, and facilitated access to data sources.

**Practice characteristics**

From the literature and expert input from our team, we listed elements of general practice care considered likely to be associated with good patient health outcomes, and lists of patient health outcomes considered potentially sensitive to models of primary care which had already been defined in the health system in Aotearoa.

We constructed a list of candidate practice characteristics from the international primary care literature (including continuity and access), from contracted literature scans specific to Māori and to Pacific interactions with primary care, and from topics we considered to be of current interest to policy-makers in Aotearoa (including the role of nurses and mental health care). The list of items went through repeated iterations with the investigators. The full list of practice characteristics sought, and the potential source of data for each, is in “Supplementary file 3 practice characteristics” (insert hyperlink).

**Practice outcomes**

We use the term patient health outcomes in the sense of variables to be predicted by regressions. These measures can also be called performance indicators measuring process and medium term outcomes. The advantage of process measures is that they are sensitive to quality of care and are a direct measure of quality [9]. The advantage of medium and long-term outcomes is that they can reflect all aspects of care including those that are otherwise difficult to measure, such as technical expertise. The disadvantage of medium and long-term outcomes, for the purpose of assessing practice performance, is that they are also affected by determinants of health other than primary care.

We decided to use measures from existing national indicator collections. These come with specifications for numerator, denominator and rationale. Lists came from: Health Quality Measures NZ; Health Quality and Safety Commission stack and Atlas of Variation; and the Health Care Home National Dataset. In all we identified more than 200 measures, including many overlaps, that might be relevant to primary care. We went through an iterative process with the investigator team to prioritise outcomes, arriving at a list of 8 primary outcomes and 8 secondary outcomes. The full list of items considered is in “Supplementary file 4 practice outcomes” (insert hyperlink).

Primary outcome measures initially planned

1. 6 month immunisation rate (timeliness)
2. ASH rates for children 0-14 (exclude elective dental)
3. ASH rates for adults 45-64
4. Polypharmacy 65+, 55+ for Māori, Pacific (5+ or 10+ to be decided after review of data)
5. ED attendance rates (all)
6. HbA1c in the previous year, for patients with diabetes
7. (Medication adherence: dropped due to complexities of calculations)
8. (Cervical smears up to date: dropped due to inconsistent data)

Secondary outcome measures

1. Immunisation 2 year rate (completeness)
2. ED attendance in daytime, low acuity
3. DNA percentage of First Specialist Assessments
4. Other immunisation analyses, flu vaccine
5. Measures from practice extracts: blood pressure, HbA1c levels, smoking ABC, CVDRA, lipids, weight, uric acid, mental health assessment score improvements
6. Disease-specific prescribing: inhaled corticosteroids, NSAIDs, BP lowering, SSRIs, tramadol, antibiotics
7. Adjustment of polypharmacy for multi-morbidity
8. Possible: “triple whammy” NSAID + ACE/ARB + diuretic

**Definitions**

*A “practice”*

We could only analyse by “practice” as reported to the national PHO dataset; only this way could we relate practice characteristics to practice-level outcomes in PHO and national data. In a small number of cases, reality was more complex. When uncertain, we enquired directly from practices and PHOs. Multiple physical practices could report as one entity for administrative and payment purposes or to meet criteria to qualify as a VLCA practice; we analysed these as a single practice. Some practices operated “satellite clinics” which functioned and reported as a single practice and we analysed them as a single practice. Finally, one venue could host multiple practices that functioned and reported independently; we analysed these as separate practices.

*Models of care*

The funding bodies issued a Request for Proposal that identified three models of care – Traditional, Corporate and Health Care Home. The research team, together with the Steering Group, recognised another four distinct models of care: PHO/DHB owned, Trust/NGO owned, Māori provider practices and Pacific provider practices.

*Traditional practice*

The funders described traditional as a model of care centred upon the general practitioner, with nursing support. We defined “Traditional” as a single practice, usually owned by GPs but could be NPs or mixed ownership with staff, run as a small business to make a profit or return income to owners. We accepted as Traditional those practices that did not obviously fit the criteria for Corporate, PHO/DHB or a Trust/NGO. It could span very small and very large organisations, and could serve high need or low need populations. Individual practices had a high degree of autonomy over service delivery. This was the longest-standing model and constituted the majority of practices.

*Corporate practice*

Corporate was defined as any group of two or more practices owned by the same for-profit business entity. We excluded groups that were clearly owned by a Trust or NGO (including Māori and Pacific practices) with an explicit health and social purpose.

A starting list was provided by NZ Doctor, a trade journal [10]. They had compiled a list of all practices they considered to be corporate, and had interviewed all the CEOs of the corporate entities. We identified a small number of additional Corporate practices from investigator knowledge, web searches and direct contact with practices.

Some Corporate practices delivered high volumes of care, with low costs for patients and often without the need for an appointment. Corporate practices had a relatively high degree of standardisation in business and clinical processes and information technology across different sites. Most Corporate practices were Traditional practices before being bought by a corporate entity.

*PHO/DHB practices*

A small number of practices were owned by a PHO or a DHB, most to continue to provide primary care services in a specific location, often an underserved and/or rural area. They did not fit our definition of Corporate, Medical Home or Traditional models of care.

*Trust/NGO practices*

These practices were owned by a not-for-profit Trust or non-governmental organisation (NGO). There was a clear and publicly-stated purpose identifying a health or social goal. Such organisations must be financially sustainable, but profit or return on investment was not a primary goal, and funding could come from multiple sources. Many were in small communities or served populations with high need. Examples included Union clinics, most Māori providers, most Pacific providers, and rural community trusts that paid staff including doctors on a salary, and may co-locate social and civic services.

*Health Care Home*

The New Zealand Health Care Home (HCD) Collaborative developed a version for Aotearoa of what was known elsewhere as the “patient-centred medical home”. The HCH maturity matrix placed specific emphasis on ready access to urgent and unplanned care; proactive care for those with more complex need; better routine and preventative care; and improved business efficiency and sustainability [11]. The HCH recognised two “stages” of HCH status: a practice was “credentialed” on initial entry to a practice development pathway and “certificated” when they had met specified standards. The Collaborative provided a list of all practices that were credentialled or certificated. At September 2018 there were 127 HCH practices of which 14 were certificated (A Maxwell, personal communication 2018); those not certificated were at different stages of meeting the maturity matrix criteria. Most had been Traditional practices prior to embarking on the HCH programme.

*Māori provider*

We sought to identify practices that would consider themselves to be Māori provider organisations in terms of ownership and governance and target population. We thought it likely that such practices offered a model of primary care that was systematically different from those offered by non-Māori provider organisations.

The Ministry of Health had no complete list of Māori provider organisations. In the year to November 2018, 33 organisations had received funding from the Māori Provider Development Fund, noting that not all potentially-eligible organisations had sought or received funding from that source in any given year.

We approached each DHB general manager or equivalent, who responded or passed on the request to the person or role in each DHB, responsible for contracting Māori primary care services or primary care services in general, and asked them for a list of practices they considered to be Māori providers or Māori organisations they contracted and which were providing primary care services. Not all responded or were able to provide such lists.

Building on the data available, we used web searches and personal contacts and advice from our Māori investigators to link contracting bodies to individual named primary care practices and map these to specific practices identified in the national PHO dataset. We could not identify a primary care practice for a small number of contracted Māori organisations; many such organisations were responsible for multiple primary care practices; and there were a small number of such practices for which we could not find a match in the national dataset.

*Pacific provider*

We sought to identify practices that would consider themselves to be Pacific providers in terms of ownership and governance and target population. We thought it likely that such practices offered a model of primary care that was systematically different from those offered by non-Pacific providers. The process we followed was the same as described above for Māori providers, including using data from the Pacific Provider Development Fund, contacting DHB general managers or contract managers, web searches, direct contact with practices or known to investigators. There may have been a small number of practices we did not successfully identify as Pacific practices.

*Ownership types*

Each practice was classified to one ownership type: Traditional, Corporate, PHO/DHB or Trust/NGO. Where practices had mixed ownership, we prioritised ownership in the following order: Corporate, Trust/Other, PHO/DHB, then Traditional. Part-ownership was common in the case of corporate entities, where an explicit model might be to share risks and benefits with part-owner GPs, NPs or other parties. Other mixed ownership examples included PHO / iwi trust, PHO / community trust, and community trust subcontracting to a corporate provider; each of these examples were classified as Trust/Other.

Importantly, HCH, Māori and Pacific practice models could overlap with ownership types. HCH practices could overlap with ownership types and Māori and Pacific practices. Māori and Pacific practices did not overlap.

*Very Low-Cost Access*

Very Low-Cost Access (VLCA) was a contract in which practices accept an increased capitation fee and guarantee a patient fee that, at September 2018, was set at a maximum of $18 for an adult. Uptake of this contract was voluntary, but to be eligible on current criteria a practice must have had an enrolled population of ≥50% patients who were “High Need”, defined as Māori, Pacific or living in an area of Quintile 5 deprivation. However, some practices with a VLCA contract no longer met the criteria current at the time of the study.

*Rural practice*

There was no agreed national or international definition of rural. We classified practices based on the rurality of a practice’s patients. The method was based around a voting classifier, where practices were initially assigned to the Statistics New Zealand category corresponding to the top meshblocks of patients enrolled in each practice. Categories were: major, satellite or independent urban; rural with high, moderate or low urban influence; or highly rural / remote. In a final step, we allocated Statistics NZ rurality classifications to Urban/Rural using the methods outlined in Fearnley et al. (2016) [12].

*Primary care clinical input*

Time spent, in the previous year, was calculated for each patient, and allocated to NP, RN, GP or Other. Other included health care assistants, dieticians, physiotherapists, Quit smoking providers and unidentified persons. The data were extracted from the appointment book and represent face to face consultations, but not telephone, email or other contacts. These data were used in the regressions.

Across most practices, nurse activity was harder to identify and analyse than GP activity. GPs tended to use only one named-provider template to record their time, which had exploitable characteristics that aided classification. For example, GP templates would consistently be coded with the GP’s Medical Council number and names nearly always began with “Dr”. Nurses, on the other hand, used both generic and named-provider templates. Even when named-provider templates were used by nurses, the practitioner profession was often not specified, making it more difficult to identify nurse activities. Generic templates were inconsistently named across practices, for example: “nurse”, “nurs”, “nur”, “N1”, and “IUCD nurse”. Many practices used a wide range of generic templates for different services, such as “diabetes clinic”.

Unclassified names on templates were cross-checked with the register of practicing nurses held by Te Kaunihera Tapuhi o Aotearoa (Nursing Council of New Zealand). Manual classification of inconsistent template names identified “hidden” nurse templates, accounting for a significant portion of nurse work in the appointment data.

Of consultations with a doctor, 82% were vocationally registered in general practice, 17% had no vocational registration (primarily doctors in a postgraduate training pathway) and 1% with other specialist vocational registration. In this study, these doctors are all referred to as GPs.

A separate calculation of Full Time Equivalent hours was calculated, at practice level, from FTE and headcount data collected from a practice survey sent by the PHOs to their practices.

*Unenrolled population*

On 30 September 2018, the NZ population was estimated to be 4,921,300 of whom 4,561,097 (92.7%) were enrolled in one of 988 general practices, leaving about 360,200 people (7.3%) not enrolled [13], depending on the accuracy of the estimated denominator. This latter group is largely outside our data collection.

Overall, non-enrolled people are less likely to have acute secondary care interactions than enrolled (G Jackson, personal communication, 2022). The main exception is 1-4 year olds, and to a lesser extent 5-9 year olds, who seek secondary care more often than enrolled patients, more for Māori and Pacific children, and those in more deprived districts. The unenrolled population may also include people at high need who seek and receive few services.

***Regression analyses***

*Data Imputation*

To address missing data we used Multiple Imputation by Chained Equations (MICE). This involves using patterns/correlations in the complete data to estimate the missing data. This approach corrects for bias from missing data and leads to accurate standard errors if the data is Missing at Random and may even produce less biased estimates when data are Missing Not At Random [14, 15].

We had complete data on most of the variables for nearly all patients (approximately 4.5 million). However, some of the PHO data were incomplete so that FTE data, for example, were available for only 540,000 (12%) of these 4.5 million patients, which could lead to biased estimates. We used a Multiple Imputation by Chained Equations procedure to estimate the missing data and increase our sample size from 540,000 to 2,500,000 patients to calculate FTE. We used the Predictive Mean Matching (PMM) procedure in R to impute the missing values for the GP FTE, Nurse FTE, NP FTE, Total Consultations, and Percent Main Provider variables. All other complete variables were used as predictors in the imputation procedure.

*Variable Selection*

A LASSO model was used to guide model specification, with final selection guided by expert opinion. LASSO models are part of a family of penalised regression algorithms which try to maximise the model’s fit while under restrictions on the number and size of the coefficients used in the model [16, 17]. We restricted the LASSO regression to choose only 10 variables. (In some cases, a larger number was required by the model). By construction, these first 10 variables are the ones most important for the model to fit the data.

The Polypharmacy, 6 Month Immunisation and HbA1c LASSO models were run on all the data relevant to each outcome, whereas the child ASH, adult ASH and ED attendances models were run on 50%, 25% and 12.5% samples of the data respectively, to avoid excessively long computation with the negative binomial regressions used for this count data.

Interaction variables were selected by observing model coefficients and reflecting on hypotheses for the cause and effect of specific variables. Interactions were generally added where clinical expertise suggested that that other variables could have a significant moderating or enhancing effect.

*Model Descriptions*

The **polypharmacy** regression included only patients who were 65 and older. The dependent variable was polypharmacy, which took value 1 if a person was taking five or more drugs and 0 otherwise. We used a logistic regression with the default glm function in R, allowing for random intercepts and random slopes on M3 and Total Consultations.

The **HbA1c** test regression included only patients on the VDR. The dependent variable was a HbA1c test, which took value 1 if a person had an HbA1c test within a year and 0 otherwise. We used a logistic regression with the default glm function in R, allowing for random intercepts and random slopes on M3, Total Consultations, and RN FTE.

The **6 month immunisations** regression included only children who were 6 months old at some point over the period of analysis. The dependent variable was 6 month immunisations, which takes value 1 if a child has had all their required immunisations (according to the vaccine schedule) by six months of age and 0 otherwise. We used a logistic regression with the default glm function in R, allowing for random intercepts and random slopes on Total Consultations, GP FTE, and RN FTE.

The **child ASH admissions** regression included only children aged 0-14 years. The dependent variable was a child’s number of ASH admissions over the analysis period. We used a negative binomial regression with the glmmTMB R package, allowing for random intercepts and random slopes on Total Consultations, GP FTE, and Percent Main Provider.

The **adult ASH admissions** regression included only adults aged 45 to 64 years old. The dependent variable was a patient’s number of ASH admissions over the analysis period. We used a negative binomial regression with the glmmTMB R package, allowing for random intercepts and random slopes on Total Consultations and M3.

The **ED attendances** regression included all patients. The dependent variable was a patient’s number of ED attendances over the analysis period. We used a negative binomial regression with the glmmTMB R package, allowing for random intercepts and random slopes for Total Consultations and M3.

**References**

1. Atkinson J, Salmond C, Crampton P: NZDep2018 Index of Deprivation, Final Research Report. Wellington: University of Otago: Wellington; 2020.

2. Jo EC, Drury PL: Development of a Virtual Diabetes Register using Information Technology in New Zealand. Healthc Inform Res 2015, 21:49-55.

3. Stanley J, Sarfati D: The new measuring multimorbidity index predicted mortality better than Charlson and Elixhauser indices among the general population. J Clin Epidemiol 2017, 92:99-110.

4. Exeter DJ, Zhao J, Crengle S, Lee A, Browne M: The New Zealand Indices of Multiple Deprivation (IMD): A new suite of indicators for social and health research in Aotearoa, New Zealand. PLoS One 2017, 12:e0181260.

5. Chan WC, Papaconstantinou D, Lee M, Telfer K, Jo E, Drury PL, Tobias M: Can administrative health utilisation data provide an accurate diabetes prevalence estimate for a geographical region? Diabetes Res Clin Pract 2018, 139:59-71.

6. New Zealand Index of Multiple Deprivation (IMD) [<https://www.fmhs.auckland.ac.nz/en/soph/about/our-departments/epidemiology-and-biostatistics/research/hgd/research-themes/imd.html>] Accessed on 9 Nov 2022

7. Salmond CE, Crampton P: Development of New Zealand's deprivation index (NZDep) and its uptake as a national policy tool. Can J Public Health 2012, 103:S7-11.

8. He ara aupiki, he ara auheke. Patient Experience Survey [<https://www.hqsc.govt.nz/our-data/patient-experience/>] Accessed on 18 Nov 2022

9. Mant J: Process versus outcome indicators in the assessment of quality of health care. Int J Qual Health Care 2001, 13:475-480.

10. Thomas F: Keeping up with the corporates: A who’s who of practice ownership In NZ Doctor; 2018.

11. Health Care Home Collaborative: Health Care Home Model of Care Requirements. Wellington: Health Care Home Collaborative; 2017.

12. Fearnley D, Lawrenson R, Nixon G: 'Poorly defined': unknown unknowns in New Zealand Rural Health. N Z Med J 2016, 129:77-81.

13. Estimated population of NZ [<https://www.stats.govt.nz/indicators/population-of-nz>] Accessed on 22 Feb 2023

14. Azur MJ, Stuart EA, Frangakis C, Leaf PJ: Multiple imputation by chained equations: what is it and how does it work? Int J Methods Psychiatr Res 2011, 20:40-49.

15. Newgard CD, Haukoos JS: Advanced statistics: missing data in clinical research--part 2: multiple imputation. Acad Emerg Med 2007, 14:669-678.

16. Buu A, Johnson NJ, Li R, Tan X: New variable selection methods for zero-inflated count data with applications to the substance abuse field. Stat Med 2011, 30:2326-2340.

17. Morozova O, Levina O, Uuskula A, Heimer R: Comparison of subset selection methods in linear regression in the context of health-related quality of life and substance abuse in Russia. BMC Med Res Methodol 2015, 15:71.
