# Supplementary material for: Is there equity of patient health outcomes across models of general practice in Aotearoa New Zealand? A national cross-sectional study
Source: Int J Equity Health. 2023 May 4;22:79. doi: 10.1186/s12939-023-01893-8 (PMC10157126; doi:10.1186/s12939-023-01893-8)
Supplement: Supplementary file 2 — Additional file 2: Supplementary file 2. Full output of final regression models. [file 12939_2023_1893_MOESM2_ESM.docx]

**Supplementary file 2. Full output of final regression models**

**Description of final regression models**

- The polypharmacy regression uses a logistic regression model. The dependent variable is polypharmacy, which takes value 1 if a person is taking five or more drugs and 0 otherwise. We allow for random intercepts and random slopes on M3 and Total Consultations.
- The HbA1c test regression uses a logistic regression model. The dependent variable is HbA1c test, which takes value 1 if a person has had an HbA1c test within a year and 0 otherwise. We allow for random intercepts and random slopes on M3, Total Consultations, and RN FTE.
- The 6 month immunisations regression uses a logistic regression model. The dependent variable is 6 month immunisations, which takes value 1 if a child has had all their required immunisations (according to the vaccine schedule) by six months of age and 0 otherwise. We allow for random intercepts and random slopes on Total Consultations, GP FTE, and RN FTE.
- The child ASH admissions regression uses a negative binomial regression model. The dependent variable is a patient’s number of ASH admissions over the analysis period. We allow for random intercepts and random slopes on Total Consultations, GP FTE, and Percent Main Provider.
- The adult ASH admissions regression uses a negative binomial regression model. The dependent variable is a patient’s number of adult ASH admissions over the analysis period. We allow for random intercepts and random slopes on Total Consultations and M3.
- The ED attendances regression uses a negative binomial regression model. The dependent variable is a patient’s number of ED attendances over the analysis period. The ED Attendances model has random intercepts and random slopes for Total Consultations and M3.

*Supplementary file 2, Table 1. Coefficients from final regression models, 924 practices*

|  | Polypharmacy, people age ≥65  N = 399,227  R^2^ = 0.364 | HbA1c, people with diabetes  N = 133,985  R^2^ = 0.1366 | Immunisations at age 6 months  N = 26,859  R^2^ = 0.0795 | Child ASH, age 0 to 14 years  N =511,845  R^2^ not applicable | Adult ASH, age 45 to 64 years  N =655,088  R^2^ not applicable | ED attendances  N =2,500,000  R^2^ not applicable |
| --- | --- | --- | --- | --- | --- | --- |
| Overall average | 38.2% | 86.9% | 75.6% | 31 per 1000 enrolled children | 38 per 1000 enrolled adults | 254 per 1000 enrolled patients |
|  | Estimate  *p* value | Estimate  *p* value | Estimate  *p* value | Estimate  *p* value | Estimate  *p* value | Estimate  *p* value |
| Intercept | -2.008843  0 *** | 1.340076  0 *** | 1.118  0 *** | -4.3729785  0 *** | -4.788318  0 *** | -2.558288  0 *** |
| *Practice models* |  |  |  |  |  |  |
| Corporate ^a^ | -0.037221  0.299250 | -0.06576  0.28913 | -0.07952  0.34745 | -0.0976413  0.24727 | 0.189563  5e-06 *** | 0.013681  0.6616 |
| PHO/DHB ^a^ | -0.122245  0.073420 | -0.02923  0.80930 | -0.03888  0.82212 | -0.1567538  0.35545 | 0.095377  0.260149 | 0.098225  0.103575 |
| Trust/NGO ^a^ | -0.004201  0.943571 | 0.154939  0.13523 | 0.234  0.09533 | 0.3240682  0.00227 ** | 0.273526  7.2e-05 *** | 0.143307  0.00383 ** |
| HCH Practice ^b^ | 0.025821  0.366038 | -0.07733  0.13418 | 0.2077  0.00100 *** | 0.0548561  0.11620 | -0.055709  0.083512 | -0.118612  5e-06 *** |
| Māori Practice ^b^ | -0.158371  0.014191 * | -0.34138  0.00241 ** | -0.6969  8.04e-07 *** | -0.00391  0.95923 | 0.056859  0.424465 | 0.091977  0.092417 |
| Pacific Practice ^b^ | -0.056913  0.580772 | -0.26497  0.10264 | -0.4501  0.03223 * | -0.0885073  0.42774 | -0.129216  0.234598 | -0.163437  0.046876 * |
| *Patient characteristics* |  |  |  |  |  |  |
| Age 00-04 |  | -4.684264  1.75e-14 *** |  | 0.2072833  0 *** |  | 0.750376  0 *** |
| Age 05-09 |  | -4.021178  0 *** |  | Reference |  | 0.159191  0 *** |
| Age 10-14 |  | -3.114613  0 *** |  | -0.9316804  0 *** |  | 0.214822  0 *** |
| Age 15-19 |  | -1.548671  0 *** |  |  |  | 0.490984  0 *** |
| Age 20-24 |  | -1.114636  0 *** |  |  |  | 0.572807  0 *** |
| Age 25-29 |  | -0.949351  0 *** |  |  |  | 0.36248  0 *** |
| Age 30-34 |  | -0.685863  0 *** |  |  |  | 0.204141  0 *** |
| Age 35-39 |  | -0.488563  0 *** |  |  |  | 0.092938  0 *** |
| Age 40-44 |  | -0.238028  7.10e-08 *** |  |  |  | 0.04525  1e-05 *** |
| Age 45-49 |  | Reference |  |  | -0.137044  0 *** | Reference |
| Age 50-54 |  | 0.163997  1.88e-05 *** |  |  | Reference | -0.014594  0.143386 |
| Age 55-59 |  | 0.330222  0 *** |  |  | 0.051897  0.021212 * | -0.085663  0 *** |
| Age 60-64 |  | 0.466111  0 *** |  |  | 0.015682  0.497387 | -0.096336  0 *** |
| Age 65-69 | -0.618659  0 *** | 0.610616  0 *** |  |  |  | -0.111715  0 *** |
| Age 70-74 | -0.269034  0 *** | 0.637178  0 *** |  |  |  | -0.048024  8e-06 *** |
| Age 75-59 | Reference | 0.50975  0 *** |  |  |  | 0.039177  0.000571 *** |
| Age 80-84 | 0.181135  0 *** | 0.321481  8.55e-13 *** |  |  |  | 0.170863  0 *** |
| Age 85+ | 0.38137  0 *** | -0.111721  0.01280 * |  |  |  | 0.414305  0 *** |
| Male ^c^ |  | 0.11015  2.34e-11 *** |  |  | 0.264069  0 *** |  |
| Māori ^c^ | -0.018257  0.313303 | 0.11015  2.34e-11 *** | -0.4375  0 *** | 0.2477083  0 *** | 0.242409  0 *** | 0.188877  0 *** |
| Pacific ^c^ | -0.178995  1.36e-11 *** | -0.13623  3.19e-06 *** | 0.0302  0.64192 | 0.3380385  0 *** | 0.247202  0 *** | 0.177801  0 *** |
| Quintile 5 ^c^ |  | -0.06583  0.00129 ** |  |  |  |  |
| IMD ^d^ | 0.115903  0 *** |  | -0.1284  2.03e-12 *** | 0.1353884  0 *** | 0.126632  0 *** | 0.097852  0 *** |
| Diabetes ^c^ | 1.463901  0 *** |  |  |  | 0.185433  0 *** |  |
| Gout ^c^ | 1.221763  0 *** | 0.228473  0 *** |  |  | 0.092033  0.005129 ** |  |
| HbA1c test ^c^ | 0.276971  0 *** |  |  |  |  |  |
| SSRI ^c^ | 0.93267  0 *** |  |  |  |  | 0.311142  0 *** |
| Tramadol ^c^ |  |  |  |  | 0.458435  0 *** | 1.007642  0 *** |
| Antibiotics ^c^ | 0.290706  0 *** | 0.207501  0 *** |  | 0.8995884  0 *** | 0.833623  0 *** | 0.621672  0 *** |
| M3 ^d^ | 0.622976  0 *** | -0.28979  0 *** |  | 2.0333836  0 *** | 1.028358  0 *** | 0.712244  0 *** |
| Continuity of practice ^d^ |  |  |  | -0.2786333  0 *** | -0.209636  0 *** | -0.226243  0 *** |
| First Specialist Assessment ^d^ | 0.092549  0 *** | 0.114845  6.49e-16 *** |  | 0.3779108  0 *** | 0.372248  0 *** | 0.379449  0 *** |
| First Specialist Assessment Did Not Attend ^d^ |  |  | -0.3893  1.27e-06 *** | 0.1442608  0.00831 ** | 0.411164  0 *** | 0.410406  0 *** |
| *Practice characteristics* |  |  |  |  |  |  |
| Urban ^c^ | -0.035963  0.198590 | -0.10261  0.04948 * | 0.1207  0.12141 | -0.0347872  0.35800 | -0.003349  0.934177 |  |
| VLCA ^c^ |  | -0.0986  0.02964 * | -0.1176  0.04088 * |  |  |  |
| Continuity of GP ^d^ |  |  |  | -0.1355638  0.00182 ** |  |  |
| Distance to nearest ED ^d^ |  |  | -0.000005357  0.00022 *** |  | -0.040168  0.00196 ** | -0.078785  0 *** |
| *Primary care clinical input* |  |  |  |  |  |  |
| NP + GP consultations ^d^ | 0.147477  0 *** | 0.08591  0 *** | 0.02519  5.91e-06 *** | 0.0837146  0 *** | 0.078097  0 *** | 0.071871  0 *** |
| RN hours ^d^ |  | 0.160711  3.21e-14 *** | 0.1224  1.93e-06 *** |  |  |  |
| GP hours ^d^ |  |  | 0.05846  0.00535 ** | -0.0055564  0.60144 |  |  |
| *Interactions* |  |  |  |  |  |  |
| NP+GP consultations X Corporate ^a^ | 0.020764  3.00e-05 *** | 0.01308  0.18051 | -0.01433  0.23311 | 0.0190497  0.00220 ** | -0.018154  6.7e-05 *** | 0.001667  0.458327 |
| NP+GP consultations X PHO/DHB ^a^ | 0.041353  4.20e-05 *** | 0.022882  0.27086 | -0.005136  0.86196 | -0.0006885  0.96852 | 0.026556  0.010997 * | 0.010033  0.032334 * |
| NP+GP consultations X Trust/NGO ^a^ | 0.021921  0.000515 *** | -0.004223  0.68653 | -0.02039  0.17478 | 0.0072696  0.34182 | -0.014969  0.005735 ** | -0.001808  0.47998 |
| RN hours X Corporate ^a^ |  | -0.125224  0.01507 * | -0.1113  0.08147 |  |  |  |
| RN hours X PHO/DHB ^a^ |  | -0.009572  0.92397 | -0.07731  0.50065 |  |  |  |
| RN hours X Trust/NGO ^a^ |  | -0.004562  0.93460 | -0.1505  0.03546 * |  |  |  |
| GP hours X Corporate ^a^ |  |  | -0.05231  0.23249 | -0.0514554  0.03855 * |  |  |
| GP hours X PHO/DHB ^a^ |  |  | -0.03456  0.74182 | 0.0466591  0.40995 |  |  |
| GP hours X Trust/NGO ^a^ |  |  | 0.001761  0.97589 | -0.039191  0.17471 |  |  |
| Continuity of GP X Corporate ^a^ |  |  |  | -0.0287257  0.76433 |  |  |
| Continuity of GP X PHO/DHB ^a^ |  |  |  | 0.2973542  0.15083 |  |  |
| Continuity of GP X Trust/NGO ^a^ |  |  |  | -0.0979557  0.37620 |  |  |

Notes: (1) Blank cells indicate variables not retained in final models. (2) *p<0.05, **p<0.01, ***p<0.001, 0*** p<1e^-16^

^a^ Reference is Traditional practice

^b^ Reference is all other practices

^c^ Dummy variable

^d^ Continuous variable
