# Supplementary material for: Is there equity of patient health outcomes across models of general practice in Aotearoa New Zealand? A national cross-sectional study
Source: Int J Equity Health. 2023 May 4;22:79. doi: 10.1186/s12939-023-01893-8 (PMC10157126; doi:10.1186/s12939-023-01893-8)
Supplement: Supplementary file 3 — Additional file 3: Supplementary file 3. Potential indicators of practice and patient characteristics. [file 12939_2023_1893_MOESM3_ESM.docx]

**Supplementary file 3. Potential indicators of practice and patient characteristics**

This list was used iteratively to guide team decisions around what indicators would be ideal and which might be available. List of tables:

- Already collated
- Basic practice descriptors
- Population attending practice, enrolled and casual
- Māori literature scan
- Pacific literature scan
- Utilisation of nurses
- Continuity
- Access
- Other markers of practice model of care
- Mental health care

The column headings include:

- ID – identification label. Gaps in ID sequence are intentional. Brackets e.g. (ID1) identifies an indicator that might measure more than one concept
- Characteristic – the target concept

Explanation

- Measures – potential data used to measure characteristic
- National data – available in national datasets
- PHO in-house data – available from Primary Health Organisations
- PHO practice extracts – available from practices via extraction by PHO
- PMS Data Elements – data element in practice management system

**Acronyms**

| CSC | Community Services Card |
| --- | --- |
| CVD | Cardiovascular disease |
| DHB | District Health Board |
| DNA | Did Not Arrive |
| EMR | Electronic Medical Record |
| FFP f | Flexible funding pool |
| FTE | Full Time Equivalent |
| GP | General Practitioner |
| HCA | Health Care Assistant |
| HCH | Health Care Home |
| HP | Health practitioner |
| Kaimahi | Community health workers |
| MECA | Multi-Employer Collective Agreement |
| MH | Mental health |
| NGO | Non-Governmental Organisation |
| NMDS | National administrative dataset, hospital admissions |
| NP | Nurse Practitioner |
| NZDep | Deprivation score assigned to all individuals residing in a small geographic area |
| PHO | Primary Health Organisation |
| PMS | Practice Management System |
| PRIMHD | National administrative dataset, mental health secondary and community care |
| ProExtra | A funding stream for primary care services |
| Q5 | Quintile 5, highest deprivation quintile of NZDep score |
| RFP | Request for Proposal |
| RN | Registered Nurse |
| RNZCGP | Royal NZ College of General Practitioners |
| Rx | Prescription |
| SIA funding | Services to Improve Access |
| SMS | Self management support |
| Te reo | Māori language |
| Tikanga | Customs and values |
| U13 / U14 | Under 13 years or Under 14 years funding contract |
| VLCA | Very Low Cost Access funding contract |
| Whanau ora | Family health |
| Whanaungatanga | Relationships |

**Already collated**

| ID | Characteristic | Explanation | Measures | National data | PHO in-house data | PHO practice extracts | PMS data Elements |
| --- | --- | --- | --- | --- | --- | --- | --- |
|  | RFP category |  | 1. Corporate 2. Medical Home 3. Traditional General Practice |  |  |  |  |
|  | HCH category |  | 1. HCH Credentialed (& date) 2. HCH Certificated (& date) 3. Not HCH |  |  |  |  |
|  | Corporate category |  | 1. Business 2. Retain GP control 3. Own model of care 4. Not corporate |  |  |  |  |
|  | PHO |  |  | PHO register |  |  |  |
|  | DHB |  |  | PHO register |  |  |  |
|  | Rural | NZ Stats / Nixon et al, “Independent Small Settlements” | 1. Urban 2. Rural |  |  |  |  |
|  | Practice size |  |  | PHO register |  |  |  |
|  | National Enrolment Service (NES) |  | Enrolled or not | NES |  |  |  |
|  | Patient Experience Survey (PES) |  | Enrolled or not | PES |  |  |  |

**Basic practice descriptors**

| ID | Characteristic | Explanation | Measures | National data | PHO in-house data | PHO practice extracts | PMS data elements |
| --- | --- | --- | --- | --- | --- | --- | --- |
| 1 | Ownership | Ownership models vary widely | 1. All GPs own 2. GP owner/s + employ GPs 3. Owned by PHO 4. Owned by non-GP company 5. Owners includes NP or RN 6. Māori provider 7. Pacific provider 8. Other ownership (e.g. NGO) |  | Ownership |  |  |
| 2 | Electronic medical record | Access may be distinctive feature of Corporate; which Electronic Medical Record (EMR) defines what capabilities are present | Which EMR; access to full clinical records by 1 or more than 1 practice |  | EMR |  |  |
| 3 | Staff | Defining FTE can be difficulty: half day clinical sessions + other clinical activity such as rest homes, school clinic, home visits | For each staff member:   1. FTE 2. Gender 3. Age 4. Ethnicity 5. vocationally registered 6. prescriber   For each profession: N per 1000 enrolled population and attending population |  | Workforce survey |  |  |
| 4 | Staff payment model | By professional group | 1. Salary 2. Hourly 3. Percent of attributed revenue 4. Mixed 5. Incentive payments |  | Payment model, MECA (multi-employer collective agreement) |  |  |
| 5 | Well established | Stable practice needed to implement and manage intended model of care; may reflect practice morale | - New GPs, NPs, Ns in last 2 years - How long individuals are present in EMR records over 5 years |  | Workforce survey | Encounter, Appointment, Service provider | Encounter: Provider  Date Time  Practitioner: Name  Registration  Qualifications  Number  ? cross reference to SCRIPT data (Script Date and Time and Script Practitioner code) to identify NPs  Cross reference to:  Invoice:  Date  Service Code |
| 6 | Accreditation | RNZCGP offers Foundation and Cornerstone accreditation | Foundation and Cornerstone status |  | Foundation, Cornerstone |  |  |
| 7 | Appointment system |  | - Books open - Appointment model:  1. Regular + ‘fit in’ urgent patients 2. Regular + ‘walk-in’ times 3. ‘Walk-in’ + planned appointments 4. Regular + acute slots kept for filling same day 5. No appointments |  | Appointment system |  | Appointment: Patient  Date Time  Practitioner  Encounter: Patient  Date Time  Practitioner |
| 8 | Opening hours | Note ‘normal’ hours for HCH considered to be 8-6 | - Opening hours  1. N hours open after 6pm weekdays 2. N hours open weekend  - After hours availability / arrangements - N hours open 8-6 weekdays |  | Hours  After-hours |  | Appointment: Date Time  Practitioner  Patient  Encounter: Date Time  Patient  Practitioner  Invoice: Patient  Date Time  Practitioner |
| 9 | Fees |  | - VLCA practice - U13 / U14 contract - Fee for adults 45-64 - Fee to see nurse - Fee for repeat prescription (options) |  | Fees |  |  |
| 10 | Languages | Languages spoken by staff; access to and use of interpreters | - Languages by staff - Interpreters |  | Languages, interpreters |  |  |

**Population attending practice, enrolled and casual**

| ID | Characteristic | Explanation | Measures | National data | PHO in-house data | PHO practice extracts | PMS data elements |
| --- | --- | --- | --- | --- | --- | --- | --- |
| 20 | Gender | Some practices may serve specific sub-populations e.g. younger women, older men | Female %; enrolled, casual attending |  |  | Gender, enrol status | Encounter: Date Time  Patient  Demographics  Registers |
| 21 | Age | Practices may serve specific sub-populations e.g. aged residential care, children & young people | Age 0-14 %; Age 65+ %; enrolled, casual attending |  |  | Age, enrol status | Encounter: Date Time  Patient Demographics  Enrolment: registers |
| 22 | Ethnicity | Māori, Pacific, Asian, NZ European / Other | Ethnic group %; enrolled, casual attending |  |  | Ethnicity, enrol status | Encounter: Date Time  Patient Demographics  Enrolment: registers |
| 23 | Deprivation | Community Service Card (CSC); NZDep Quintile | CSC %, NZ Dep Q5 %; enrolled, casual attending |  |  | NZDep, CSC, enrol status | Encounter: Date Time  Patient Demographics  Enrolment: registers |
| 24 | High Needs | High Needs = Māori or Pacific or NZDep Q5 | High Needs %; enrolled, casual attending |  |  | High Needs, enrol status | Encounter: Date Time  Patient Demographics  Enrolment: registers |

**Māori literature scan**

| ID | Characteristic | Explanation | Measures | National data | PHO in-house data | PHO data extracts | PMS data elements |
| --- | --- | --- | --- | --- | --- | --- | --- |
|  | Ownership | Māori provider owned (data may come from DHBs) |  |  | (ID1) |  |  |
|  | Equity | All data to be cut by ethnicity |  |  |  |  |  |
|  | Access | Able to get appointment at a manageable time |  |  | (ID7, ID8) |  |  |
|  | Costs | Economic costs (data not collected on transport, childcare, fees, time off work) | - Practice fees - Prescription fees; use national policies for cost rules | Ethnicity, CSC, NZDep | (ID9) |  |  |
|  | Response to Costs | Practice, PHO or DHB response to costs e.g. ProExtra, SiA-funded services | - Transport / petrol / parking - Childcare - Links to social services |  | (ID62)  Cost reduction |  |  |
|  | Support services | Financial, childcare, emotional support;  Kaimahi (community health workers, navigators) advocating and navigating health and social services | Kaimahi  Whanau Ora contract |  | (ID3)  Outreach contracts; MH programmes |  |  |
|  | Distance | Geographic distance |  |  | (ID63, ID55, ID64) |  |  |
|  | Cultural safety | Training programmes available; Eurocentric practices a barrier. Te Ao Māori practices (tikanga, te reo Māori, wh$ā$nau centered) facilitate access through sense of comfort, empowerment, cultural acceptability, belonging | Attendance, completion by individuals or profession |  | Cultural training |  |  |
|  | Home visits |  |  |  | (ID54) |  |  |
|  | Whanaungatanga | Relationships /engagement / rapport; lack of trust in GPs / HPs; treated with respect, non-judgemental; treat people as a whānau rather than individual patients; listening to patients; racism and other forms of discrimination (body weight, place of residence); shared decision-making. |  | Pt Experience Survey |  |  |  |
|  | Ethnic concordance | Preference for HPs of the same ethnicity (shared language, understandings of the world) | Ethnic concordance by visit, by profession |  | (ID3, ID41) |  |  |
|  | Continuity of care | Long term relationships with GP/HCPs |  | Churn | (ID32) |  |  |
|  | Health literacy | Medical jargon, need culturally appropriate communication / resources, preference for visual |  | Pt Experience Survey |  |  |  |
|  | Languages |  |  |  | (ID10) |  |  |
|  | Missed services | May mark patient unmet need and barriers to service access | DNA at primary and secondary care | DNA |  |  | (ID59) |

**Pacific literature scan**

| ID | Characteristic | Explanation | Categories / measures | National data | PHO in-house data | PHO practice extracts | PMS data elements |
| --- | --- | --- | --- | --- | --- | --- | --- |
|  | Ownership | Pacific provider owned (data may come from DHBs) |  |  | (ID1) |  |  |
|  | Equity | All data to be cut by ethnicity |  |  |  |  |  |
|  | Access | Practice factors (data not collected on role of receptionists, practice environment, employment circumstances)  Barriers to access: | - opening hours - appointment system |  | (ID7, ID8, ID75) |  | Appointment: Date Time  Patient  Demographics |
|  | Costs | Cost to see primary care, prescriptions | - Practice fees - Prescription fees; use national policies for cost rules | Ethnicity, CSC, NZDep | (ID9) |  | Prescribing: Date Time  Medication  Patient  Practitioner |
|  | Support services | Culture-specific contracts or referrals to such services | Outreach contracts  Culture-specific contracts |  | Outreach contracts |  |  |
|  | Distance | Geocode distance to practice, hospital? |  |  | (ID63, ID55, ID64) |  | Enrolment:  Patient geocode  Address  demographics |
|  | Ethnic concordance | Preference for HPs of the same ethnicity (shared language, understandings of the world) | Ethnic concordance by visit, by profession |  | (ID3, ID41) |  |  |
|  | Cultural safety | Training programmes available; Pacific models of care; Traditional health beliefs, spirituality, use of traditional healing;  Family rather than individual approach to health, support role of family in navigating health systems, family networks as source of health information, family involvement in decision making; Care of parents, elders | Attendance, completion by individuals or profession |  | Cultural training |  |  |
|  | Respect and trust | Relationships /engagement / rapport; lack of trust in GPs / HPs; treated with respect, non-judgemental; treat people as a whānau rather than individual patients; listening to patients; shared decision-making; Prior experience; Discrimination (attitudes of staff), Power differences health professionals / patients, traditionally HP held in high esteem |  | Pt Experience Survey |  |  |  |
|  | Length of consultations | Positive relationships require HP to take time, this can lead to engagement and improved adherence. | Consultation length |  |  | (ID61) | Appointment: Date Time  Practitioner  Patient |
|  | Pacific workforce |  |  |  | (ID3) |  |  |
|  | Health literacy | Medical jargon, need culturally appropriate communication / resources, preference for visual |  | Pt Experience Survey |  |  |  |
|  | Languages |  |  |  | (ID10) |  |  |
|  | Fragmented services | Lack of coordination, referral through primary care to specialist services Primary and secondary services, referrals, support services. |  |  | (ID31, ID51) |  | Outbox:  Date Time  Patient  Practitioner  Demographics |
|  | Broader community needs | Contracts addressing broader community needs (gardens, child care centres, homework programme, truancy, family violence, fire and police and community governance / ownership |  | OIA for social contracts with PHOs? |  |  |  |

**Utilisation of nurses**

|  | Characteristics | Explanation | Measures | National data | PHO in-house data | PHO practice extracts | PMS data elements |
| --- | --- | --- | --- | --- | --- | --- | --- |
| 70 | Nurses : doctors |  |  |  | (ID3) |  |  |
| 71 | Nurse PHO programmes | Nurse-relevant programmes from PHO/DHB vary across country |  |  | (ID51) |  |  |
| 72 | Nurse clinics | Acute / chronic / for what population; clinical case management | Practice-specific clinics and roles |  | clinics |  |  |
| 73 | Support health care assistant | Allows nurse to work consistently at higher scope | Presence of HCA in practice |  | (ID3) |  |  |
| 74 | Appointments | Supportive appointment system gives protected time | % appointments NP/N/GP, by disease |  | (ID7) | Appointments, profession, Read | Appointment: Date Time Practitioner  Patient |
| 75 | Screening | Primary prevention, health promotion | Cardiovascular (CVD) / diabetes screening cut by profession | Immunisation, screening rates |  |  | Recall:  Date Time  Practitioner  Patient  Demographic  Contact indicator  Appointment: Clinical Measure  Date Time  Provider  Patient  Values  [Clinical Measures Lookup Data] |
| 76 | Post-graduate education | Number of nurses with post-graduate education, financial and release time support for post-graduate education and other professional development | Nurse post-grad education; PHO/DHB support for post-grad education |  | PHO/DHB nurse support programmes |  |  |
| 77 | Nurse prescribing | Marker of high scope nursing role | Health conditions, level of independence  % Rx by nurse, by disease |  | (ID3)  Nurse prescriber role | Prescriber, profession. Read | Prescription: Date Time  Medication  Practitioner  Patient  Clinical Code: Patient  Long Term |
| 78 | Nurse Practitioner | Role in practice | NP role  % Rx (prescriptions) by NP, by disease |  | (ID1, ID3, ID4)  NP role | Prescriber, profession, Read | Prescription: Date Time  Medication  Practitioner  Patient  Clinical Code: Patient  Long Term |
| 79 | Leadership & competency | Leadership responsibility, management responsibility, recruitment and support of new graduates, line accountability for nurses; membership of a prof organisation and therefore the ownership of indemnity insurance - NZNO or CANZ automatically insured | PDP Prof Development Portfolio  PDRP Prof Development Recognition Programme |  | PDP  PDRP  Membership of prof organisation |  |  |
| 80 | Teamwork |  |  |  | (ID3, ID30, ID31) |  |  |

**Continuity**

| ID | Characteristic | Explanation | Measures | National data | PHO in-house data | PHO practice extracts | PMS data elements |
| --- | --- | --- | --- | --- | --- | --- | --- |
| 30 | Informational continuity | Information underpins safe and appropriate care | EMR capabilities; range of sources incoming information and referral destination counts |  | (ID2) | Inbox and outbox source & destination | Inbox:  Date Time  Subject  Subject Code  Abnormal Flag  External Application  External Reference  Outbox:  Date Time  Subject  Practitioner  Patient  Document Code |
| 31 | Management continuity | Guidelines / pathways; PHO/DHB supported programmes often have template for care plan | Guidelines, active and passive pathways; describe integration into EMR |  | (ID51)  Guideline / pathway |  |  |
| 32 | Relational Continuity | An ongoing therapeutic relationship between a patient and one or more providers; appointments on a future day may be by choice to see a specific provider | % visits with same provider; number of providers of same professional group seen in time period; % appointments to see Own provider same day and later day |  |  | Service provider, usual provider, by consult | Encounter: Date Time  Provider  Patient  Appointment: Date Time  Practitioner  Patient  Provider Registration Number  Provider Qualifications  Invoice:  Date Time  Patient  Practitioner  Service Code |
|  | Churn | Churn inhibits continuity, may increase cost as a casual patient or new patient enrolling at next practice; may reflect mobility of population and transient housing | By age, ethnicity; effected by practice size, geography  Change of practice with and without change of address?  Patients enrolled in one practice seeing another (Fee for Service clawback) | PHO register, General Medical Subsidy (GMS) collection |  |  | Encounter: Patient  Appointment: Date Time  Practitioner  Patient  Provider Registration Number  Provider Qualifications  Invoice:  Date Time  Patient  Practitioner  Service Code |

**Access**

| ID | Characteristic | Explanation | Measures | National data | PHO in-house data | PHO practice extracts | PMS data elements |
| --- | --- | --- | --- | --- | --- | --- | --- |
| 40 | Approachability | No data collected (Transparency, Outreach, Information, Screening) |  | Unenrolled patients |  |  |  |
| 41 | Acceptability | Professional values, norms, culture, gender | Female GP available; Male GP available; concordance patient gender, ethnicity |  | (ID3) | Gender, ethnicity, per consult | Appointment: Date Time  Patient  Provider  Encounter: Date Time  Provider Demographics |
| 42 | Availability | Hours available, appointments model | Appointments same day at 9am, 12 midday, 8am following day, 2 weeks |  | (ID7, ID8) | Appointments | Appointment: Date Time  Patient  Provider |
| 43 | Affordability | Fees (don’t have measures of other costs) |  |  | (ID9) |  |  |
| 44 | Appropriateness | See clinical quality markers |  |  |  |  |  |

**Other markers of practice model of care**

| ID | Characteristic | Explanation | Measures | National data | PHO in-house data | PHO  practice extracts | PMS data elements |
| --- | --- | --- | --- | --- | --- | --- | --- |
| 50 | PHO/DHB support | Examples: technology and business facilitators; GP and Nurse education; employ community health workers, podiatrist, pharmacist | PHO support to practices |  | Support type |  |  |
| 51 | PHO/DHB programmes | Examples: Primary Options for Acute Care (POAC), Advance Care Plan, Care Plus, Planned Proactive Care, Diabetes, chronic condition management, mental health, SIA/High Needs, palliative care, Care Plus, outreach into schools; many via FFP Flexible Funding Pool | - FFP bulk funded? - Identify programmes, activity counts and other existing PHO measures - nurse-relevant? |  | Programmes, existing PHO measures |  |  |
| 52 | Teamwork | Main markers are opportunities for communication | - Number of professions - Practice meetings, how often, who comes - Same-day appointments 2 or more professions |  | (ID3)  practice meetings | Profession, appointment |  |
| 53 | Referrals | Comprehensiveness; team roles and responsibility | Referral rates by destination, profession of referrer |  |  | (ID30)  profession |  |
| 54 | Home visit | May be practice driven or PHO support service | Which programme / fund; which profession |  | Home visits |  |  |
| 55 | Portal | Patient portals.  TF: all this information is required to be reported to MoH | Which portals; % patients enrolled; profile of use purpose, profile of use type |  | Use of portal | Use of portal | Portal: Patient  Inbox Subject  Inbox Type |
| 56 | Alternatives to face-to-face consults | Virtual consults, email, phone, technologies appropriate to rural and younger populations | Telemedicine, virtual consult, communication via email, txt or portal |  | Alternative consults |  | Portal:  Inbox Type  Appointment |
| 57 | Triage | Systematic triage can offer alternatives to face-to-face consultations; who does triage?. Note GP triage is a feature of HCH | - None - Nurse-only - NP and GP - Nurse and GP - GP only - Team triage |  | triage |  | [maybe from service codes if being used… but unlikely] |
| 58 | Self-management support (SMS) | Provision of and referrals to self-management support | Contracts for SMS; health coach or community health worker available |  | Contracts for SMS, health coach, community health worker |  |  |
| 59 | DNA rates | DNA at general practice appointment may mark patient unmet need and is disruptive to practice | DNA rates, profile of persons who DNA compared to practice profile |  | Response to DNA | Missed appointments | Appointment: Date Time  Patient  Arrived Flag  Encounter:  Date Time  Provider  Prescription: Patient  Prescription Provider  Invoice:  Date Time  Provider |
| 60 | Frequent attenders | May be a specific target for management | % enrolled population attending more than 10x in a year; what proportion attend 10x in following year |  |  | Attendance | Encounter: Date Time  Patient  Practitioner  Demographics  Appointment: Date Time  Patient  Provider |
| 61 | Length of consultations | Longer consultations may be associated with appropriate response to culture, medical complexity and social needs;  TF: may be reflected in FFP budgets of practices | Appointment length, profile of population with longer or shorter appointments |  |  | Appointments | Appointment: Date Time  Length  Patient  Provider  Practitioner Registration  Practitioner Qualifications |
| 62 | Discounts | Discount is one response to low patient income; discount may also be due to brief or repeat service; TF: may be reflected in the FFP budgets of practices | % and population profile of patients receiving discount |  |  | Actual v usual fee | [Could capture service fees in invoice data, but (a) this will be sensitive to practices, plus (b) will still be hard to identify discounting] |
| 63 | e-Resources | Via website or portal, practice or PHO | e-resources |  | e-resources |  |  |
| 64 | Distance | Geocode distance NMDS address to practice or hospital? | - Practice - Hospital | Geocode? |  |  | Patient Demographics |

**Mental Health care**

| ID | Characteristic | Explanation | Measures | National | PHO in-house data | PHO practice extracts | PMS data elements |
| --- | --- | --- | --- | --- | --- | --- | --- |
|  | Mental health patients | Mental health diagnoses in PRIMHD or inpatient or outpatient data; Read codes from primary care. Descriptive and exploratory only | Broad diagnostic categories | ICD10 codes |  | Read codes | Clinical Code:  Clinical Code Date Time  Long Term |
|  | Psychotropic medications | Antidepressant Rx (esp under 12; 13-18 years; 19-25 years. Descriptive / talking point; with non-pharm services available expect Rx rate down; so PHO support relevant; collect description of local access to psych services incl private clin psych / counsellors | Dispensing rates by age, ethnicity, gender | Pharms | Description of PHO support and local services available | Prescription rates by profession | Prescribing: Date Time  Practitioner  Medication |
|  | Atypical antipsychotics, oral | Sleep, anxiety, mood disorders; Describe, eg management of small n of severe pts, and off-label Rx esp rest-home; PHO/DHB special projects eg metabolic monitoring | Dispensing rates by age, ethnicity, gender |  | PHO/DHB special projects | Prescription rates by profession | Prescribing: Date Time  Practitioner  Medication  Demographics  Enrolment registers |
|  | Atypical antipsychotics, depot  Consider dropping | Consider correlation with status under the Mental Health Act; N too small; except if NP is central; even then may just describe a specialist service with or without integration primary care / secondary care | Dispensing rates by age, ethnicity, gender | Pharms | NP role | Prescription rates by profession | Prescribing: Date Time  Practitioner  Medication  Demographics  Enrolment registers |
|  | Referrals to secondary MHS | In PRIMHD, inpatient, want to know local secondary service model; Descriptive; map against DHB and PHO variation | Referral rates and practitioner | PRIMHD | Describe local service | Referral rates by profession |  |
|  | Referrals to counsellors and other services | Describe |  |  | Describe PHO psych services |  |  |
|  | RN/NP appointments MH patients | RN may see for depot injections, metabolic monitoring; describe | N appointment rate for MH patients, with and without co-morbidities |  |  | Appts by profession |  |
|  | ADHD | Local PHO/DHB model for ADHD / Ritalin; describe; but complex |  |  | Known to PHO as ADHB special interest practice? | % prescribing by individual in practice; prescribing to patients enrolled elsewhere | Prescribing: Date Time  Practitioner  Medication  Demographics  Enrolment registers |
|  | Alcohol, recreational drugs | Likely to be special case enthusiast practices | PHO screening and coding requirement |  | Known to PHO as special interest practice? | Read coding |  |
|  | Physical health management | Equally-well; management of physical health in pts with MH disorder | CVD / diabetes risk assessment in those with and without mental health diagnoses |  |  | CVD / diabetes risk assess with and without MH diagnosis |  |
