# Supplementary material for: Is there equity of patient health outcomes across models of general practice in Aotearoa New Zealand? A national cross-sectional study
Source: Int J Equity Health. 2023 May 4;22:79. doi: 10.1186/s12939-023-01893-8 (PMC10157126; doi:10.1186/s12939-023-01893-8)
Supplement: Supplementary file 4 — Additional file 4: Supplementary file 4. Potential practice outcome measures and sources of data. [file 12939_2023_1893_MOESM4_ESM.docx]

**Supplementary file 4. Potential practice outcome measures and sources of data**

These indicators come from searching New Zealand national indicator collections for existing indicators already defined by numerator, denominator and rationale. Items marked ‘Yes’ under Retained round 1 and 2 represent stages of the iterative prioritisation undertaken with our team of investigators. Final selections were made after assessing availability and quality of data.

Lists here are from:

- The Health, Safety and Quality Commission Measures Library | He Kete Rauemi

https://www.hqsc.govt.nz/our-data/measures-library/ (accessed 21 Nov 2022)

- Health Care Home National Dataset

https://healthcarehome.org.nz/download/health-care-home-model-of-care.pdf (accessed 21 Nov 2022)

**Acronyms**

| ASH | Ambulatory Sensitive Hospitalisation |
| --- | --- |
| DHB | District Health Board |
| DNA | Did Not Arrive |
| ED | Emergency Department |
| FSA | First Specialist Assessment |
| GP | General Practitioner |
| HCH | Health Care Home |
| NZDep | Deprivation score assigned to all individuals residing in a small geographic area |
| PHO | Primary Health Organisation |
| Q5 | Quintile 5, highest deprivation quintile of NZDep score |
| VLCA | Very Low Cost Access funding contract |

**Health Care Home National Dataset measures**

|  |  | Retained round 1 | Retained round 2 | Comment |
| --- | --- | --- | --- | --- |
| 1 | Age standardised ED attendances per 1000 enrolled patients | Yes |  | Could try analysing ED Triage 4/5 (lowest priority) in work hours separately |
| 2 | Age standardised After Hours Consultations per 1000 enrolled patients | Yes | Yes | Is this consults after 5 pm which could be routine visits during extended hours or is it consults after 5 pm at another location? |
| 3 | Age standardised ASH Admissions per 1000 enrolled patients | Yes | Yes |  |
| 4 | Age standardised Acute Admissions & readmissions per 1000 enrolled patients | Yes |  |  |
| 5 | Triage outcomes % of patients managed appropriately without a same day face to face appointment | No |  | Indici practices can show this (Electronic Medical Record software)  should be avail for Health Care Homes |
| 6 | Age standardised After Hours primary care Consultations per 1000 enrolled patients | Yes |  | How does this differ from 2? |
| 7 | Primary options for acute care claim volumes per 1000 enrolled population | Yes |  | PHO data  funding varies between PHOs |
| 8 | Same day access for those where clinically appropriate | Yes |  | Practice data  difficult to obtain comparable data |
| 9 | Accident and Medical (A&M) / other Practice visits during business hours | Yes |  |  |
| 10 | Hospital bed days in the last 6 months of life | Yes |  | Depends on Aged Residential Care (ARC) availability |
| 11 | Average patient wait time to consult | Yes |  | Part covered elsewhere |
| 12 | Annual audit of triage patients and re presentations | ? |  |  |
| 13 | Age standardised Nurse Consultations per 1000 enrolled patients | Yes |  | Part covered elsewhere |
|  | Age standardised GP consultations per 1000 enrolled patients |  |  |  |
| 14 | Percentage of patients seeing their own GP | Yes |  | Part covered elsewhere (registering with a ‘usual GP’ varies between practices) |
| 15 | Average number of different clinicians seen over the last 10 visits | Yes |  | Part covered elsewhere (teamwork v continuity) |
| 16 | BMJ measure: percentage of consults with the GP seen most often over the 24month period | Yes |  | Part covered elsewhere |
| 17 | Percentage of DNAs at hospital FSAs | Yes |  |  |
|  | Referral rates – age-std rate of (FSAs + DNAs+ ?declined) | Yes |  | Not sure ‘declined’ available everywhere |
| 18 | Partners in Health Scale — change in average score over time | Yes |  | PHO data |
| 19 | % of high needs patients with a care plan and named coordinator | Yes |  | PHO data (high needs can be clinical or defined as Māori or Pacific or living in Quintile 5 deprivation area). Process measure |
| 20 | Number of patient inbound secure messages through patient portal/1000 adults | Yes |  | Indici (software) has data)  Manage My Health count of inbound messages available |
| 21 | No. of virtual (telephone/video) planned consults as % total consults | ? |  | Indici yes  Manage My Health yes |
| 22 | Patients with activated patient portal access per enrolled population | Yes |  |  |
| 23 | % of patients that have access to own notes (PHO measure) | ? |  | Manage My Health yes |
| 24 | Smoking quit rate | ? |  | Quit rate may be determined with established interval; patient changing practices complicates |
| 25 | Dropped call rate | No |  | Pinnacle (PHO) practices using call centre can measure this |
| 26 | Patient experience survey scores | No |  | Covered elsewhere |
| 27 | Wait times in the practice (post appointment time) | No |  | Covered elsewhere |
| 28 | Time to 3rd available appointment | Yes |  | Practice extract |
| 29 | Percentage of DNAs at the practice | Yes |  | Practice extract |
| 30 | Practice team climate survey results | No |  | Data not available  Practice team climate survey (required for Planned Proactive Care programme at South Auckland DHB) |
| 31 | % Room utilisation for clinical interactions | No |  | Data not available  Could we ask about % room utilisation? |
| 32 | No of aged standardised patients enrolled per GP Full Time Equivalent (FTE) | Yes |  |  |
| 33 | No of aged-standardised patients enrolled per Nurse/ FTE | Yes |  |  |
| 34 | % of enrolled population who leave during the year | Yes |  |  |
| 35 | Staff turnover | No |  | Covered elsewhere |
| 36 | Sick days per FTE per year | No |  | Covered elsewhere |
| 37 | Total phone calls per 1000 per month | No |  | Covered elsewhere |

**Health Quality Measures NZ**

https://www.hqsc.govt.nz/our-data/measures-library/

|  | SYSTEM LEVEL MEASURES – exclusively secondary care removed | Retained round 1 | Retained round 2 | Comment |
| --- | --- | --- | --- | --- |
|  | **Childhood Ambulatory Sensitive Hospitalisations** |  |  |  |
|  | Ambulatory Sensitive Hospitalisations Dental | No |  |  |
|  | Ambulatory Sensitive Hospitalisations Maternal | No |  |  |
|  | Ambulatory Sensitive Hospitalisations Other |  |  |  |
|  | - B4 school checks are started before children are 4 1/2 years old | No |  |  |
|  | - Children with body mass index 98th percentile referred | ? |  |  |
|  | - Four year old children who have received a B4 School Check | ?Yes |  |  |
|  | - Infants who have received all WCTO core contacts due in their first year | No |  |  |
|  | Ambulatory Sensitive Hospitalisations Respiratory |  |  |  |
|  | - Hospital admissions for children aged five years with a primary diagnosis of asthma | Yes | Yes | Consider eczema and cellulitis |
|  | Ambulatory Sensitive Hospitalisations Tobacco Control |  |  |  |
|  | - Four year old children living in a smokefree home | ?No |  |  |
|  | Ambulatory Sensitive Hospitalisations Vaccine Preventable Diseases |  |  |  |
|  | - Children fully immunised by eight months | Yes |  | ?only 1 of these |
|  | - Children fully immunised by five years | Yes |  |  |
|  | - Children fully immunised by two years | Yes |  |  |
|  | **Acute Hospital Bed Days** |  |  |  |
|  | Acute Hospital Bed Days Admissions |  |  |  |
|  | - Acute readmissions to hospital | Yes |  |  |
|  | - Acute/arranged hospital admissions of PHO enrolled population aged 15 to 74 years | Yes | Yes |  |
|  | - Acute/arranged hospital admissions of PHO enrolled population aged 29 days to 14 years | Yes | Yes |  |
|  | - Ambulatory Sensitive Hospitalisation (ASH) rate per 100,000 population for 45 to 64 year olds | Yes | Yes | Less sensitive as get older, different definitions |
|  | - Ambulatory sensitive hospitalisation rate per 100,000 population for 0 to 4 year olds | Yes | Yes |  |
|  | - Inpatient Average Length Of Stay (ALOS) for acute admissions | Yes |  |  |
|  | - Inpatient average length of stay for acute readmissions for people aged 20 to 64 years old | Yes |  |  |
|  | - Occupied bed days for patients 75 years and over who had two or more emergency admissions within a calendar year | Yes |  |  |
|  | Acute Hospital Bed Days Cardiovascular/Diabetes |  |  |  |
|  | - HbA1c test results; [Percentage or number of enrolled people in the PHO within the eligible population with a record of a Diabetes Annual Review during the reporting period whose HbA1c test result is 64mmol/mol or less] | Yes |  |  |
|  | - PHO enrolled people within the eligible population who have had a cardiovascular disease (CVD) risk recorded within the last five years | Yes |  | Would make it 10 year risk |
|  | Acute Hospital Bed Days Falls and Fractures |  |  |  |
|  | - Over 65 hospital admission for falls | No? |  |  |
|  | Acute Hospital Bed Days Mental Health | No |  | Numbers too small |
|  | Acute Hospital Bed Days Other |  |  |  |
|  | - Patients admitted, discharged, or transferred from an emergency department within six hours | No |  |  |
|  | Acute Hospital Bed Days Polypharmacy |  |  |  |
|  | - People aged 65 years and over dispensed 11 or more unique long term medications | Yes | Yes | Want just 1?  Consider terms polypharmacy, hyper-polypharmacy |
|  | - People aged 65 years and over dispensed eight, nine or ten unique long term medications | Yes | Yes |  |
|  | - People aged 65 years and over dispensed five or more unique long term medications | Yes |  |  |
|  | - People aged 65 years and over dispensed five, six or seven unique long term medications | Yes |  |  |
|  | Acute Hospital Bed Days Tobacco Control |  |  |  |
|  | - Better help for smokers to quit (PHO) ; [Percentage or number of PHO enrolled patients who smoke have been offered help to quit smoking by a health care practitioner in the last 15 months] | Yes |  | Need % as VLCA have much bigger numbers |
|  | - Mothers who are smokefree at two weeks post-natal | No |  | Should be 6 week |
|  | - PHO enrolled patients who smoke have been offered help to quit smoking by a health care practitioner in the last 15 months | Yes |  | No measure of % practice population who smoke – could add |
|  | - Pregnant women who identify as smokers upon registration | No |  |  |
|  | Acute Hospital Bed Days Tobacco Control |  |  |  |
|  | - Better help for smokers to quit (PHO) ; [Percentage or number of PHO enrolled patients who smoke have been offered help to quit smoking by a health care practitioner in the last 15 months] | Yes |  | Duplicate |
|  | - Hospital patients who smoke and are seen by a health practitioner in a public hospital are offered brief advice and support to quit smoking | No |  |  |
|  | - Mothers who are smokefree at two weeks post-natal | No |  | Duplicate |
|  | - PHO enrolled patients who smoke have been offered help to quit smoking by a health care practitioner in the last 15 months | Yes |  | Duplicate |
|  | - Pregnant women who identify as smokers upon registration | No |  | Duplicate |
|  | Acute Hospital Bed Days Vaccine Preventable Diseases |  |  |  |
|  | - Influenza vaccinations for 65 year olds and over | Yes |  |  |
|  | **Patient Experience of Care** |  |  |  |
|  | Patient Experience of Care Primary Care Services | No |  | Covered elsewhere |
|  | Patient Experience of Care Secondary Care Services | No |  |  |
|  | **Amenable Mortality** |  |  |  |
|  | Amenable Mortality Cancers |  |  |  |
|  | - Breast Cancer Screening Coverage (45-69) | Yes |  | Just one will predict the others |
|  | - Breast Cancer Screening Coverage (50-64) | Yes |  |  |
|  | - Breast Cancer Screening Coverage (50-69) | Yes | Yes |  |
|  | - Women enrolled in a PHO aged 25 to 69 years who have had a cervical sample taken in the past three years | Yes | Yes |  |
|  | Amenable Mortality Cardiovascular |  |  |  |
|  | - Ischaemic cardiovascular disease; [Percentage or number of enrolled people in the PHO within the eligible population who have Ischaemic cardiovascular disease] | Yes |  |  |
|  | - PHO enrolled people within the eligible population who have had a CVD risk recorded within the last five years | Yes |  |  |
|  | Amenable Mortality Diabetes |  |  |  |
|  | - Diabetes Detection | Yes |  |  |
|  | - Diabetes Detection and Follow Up | Yes |  |  |
|  | - HbA1c test results | Yes |  |  |
|  | - Identification of Microalbuminuria in People with Diabetes | Yes | Yes | Practice extract |
|  | - Improved Management of long term conditions (Diabetes); [Primary Health Organisation (PHO) enrolled people aged 15 to 74 years with diabetes by most recent HbA1c level within the past 12 months] | Yes | Yes | Target is 100% per year for HbA1c |
|  | Amenable Mortality Other |  |  |  |
|  | - Children with BMI greater than 98 percentile referred (excluding advice given)   [Data Sources: B4 School Check data set; Primary Health Organisation (PHO) Enrolment Register] | ?No |  | Is there a measure of child obesity as such?  Children aged 4 – 15 years who have height and weight recorded and BMI classified within last year. |
|  | - Youth Suicides | No |  | Numbers too small |
|  | Amenable Mortality Tobacco control |  |  |  |
|  | - Better help for smokers to quit (PHO) | Yes |  | Duplicate |
|  | - Pregnant women who identify as smokers upon registration | No |  | Duplicate |
|  | Amenable Mortality Vaccine preventable diseases |  |  |  |
|  | - Children aged eight months are fully immunised | Yes |  | Choose 1 |
|  | - Children aged two years are fully immunised | Yes |  |  |
|  | - Children fully immunised by five years | Yes |  |  |
|  | - Primary Health Organisation (PHO) enrolled people aged 65 years and over who have received an influenza vaccination during the most recent influenza campaign | Yes |  | Duplicate |
|  | **Babies Living in Smokefree Homes** |  |  |  |
|  | Babies Living in Smokefree Homes LMC Services | No |  | Or smokefree homes at 2 weeks |
|  | Babies Living in Smokefree Homes Primary Care Services |  |  |  |
|  | - Better help for smokers to quit (PHO) | Yes |  | Duplicate |
|  | - Newborns enrolled in a Primary Health Organisation by three months | Yes | Yes |  |
|  | - PHO enrolled patients who smoke have been offered help to quit smoking by a health care practitioner in the last 15 months | Yes |  | Duplicate? |
|  | - Pregnant women registered with a Lead Maternity Carer within first trimester of pregnancy | ?No |  |  |
|  | Babies Living in Smokefree Homes Secondary Care Services | No |  |  |
|  | Babies Living in Smokefree Homes Well Child Tamariki Ora Services | No |  |  |
|  | **Youth access to and utilisation of youth appropriate health services** |  |  |  |
|  | Youth Access to Health Clinical |  |  |  |
|  | - Contraceptive Dispensing | Yes |  |  |
|  | - Positive HoNOSCA Change; [Health of the Nation Outcome Scale Child and Adolescent] | No |  | Data not available |
|  | - Utilisation of Primary Health Services by Youth | Yes | Yes |  |
|  | - Youth Accessing Timely Specialist Services |  |  |  |
|  | Youth Access to Health Community |  |  |  |
|  | - Score of 12 or more on K-10 | No |  | Data not available |
|  | Youth Access to Health Population |  |  |  |
|  | - Diagnoses of Common Mental Disorder | No |  | Data not available |
|  | - ED Presentations Resulting from Deliberate Self Harm | ?yes |  | Coding accurate? Is heavy alcohol intake deliberate self-harm ? |
|  | - Girls Fully Immunised with HPV Vaccine | Yes | Yes | By age 26?  Could be hard to measure Fully immunised, maybe count if had at least 1 dose  Maybe better Youth fully immunised by age 15  Boys? |
|  | - Oral Health - DMFT score at Year 8 | No |  | Data not available |
|  | - Unmet Need Reported By Youth; [Data Sources: Patient satisfaction survey results; Child and adolescent mental health service (CAMHS) data; PHO enrolment registers; PHO service utilisation reports; NZ health survey | Yes |  | Partial |
|  | - Utilisation of Dental Services by Adolescents | No |  | Cut by location, socioeconomic status and ethnicity. May be alternative to youth health primary car service utilisation |
|  | - Youth Suicides | No |  | Duplicate |

|  | HEALTH QUALITY AND SAFETY COMMISSION STACK – secondary care measures & duplicates excluded | Retained round 1 | Retained round 2 | Comment |
| --- | --- | --- | --- | --- |
|  | - In-hospital Associated with Fractured Neck of Femur | No? |  |  |
|  | - Hospital Days During the Last Six Months of Life | Yes |  | Duplicate |
|  | - Hospital Unplanned and Unexpected Readmission Rates | Yes? |  |  |
|  | - Mental Health Readmission % - 28 Day Acute Inpatient Readmission Rate | No |  | Numbers too small |
|  | - Occupied Bed-Days for People Aged 75+ Admitted as an Emergency 2 Times or More per Year | Yes? |  | Duplicate |

|  | LONG TERM CONDITIONS STACK – duplicates removed | Retained round 1 | Retained round 2 | Comment |
| --- | --- | --- | --- | --- |
|  | - Delay of Progression of Diabetic Nephropathy | Yes |  | Practice extract |
|  | - Monitoring Oral Anticoagulation in Primary Care | Yes |  | Practice extract |
|  | - Managing Oral Anticoagulation in Primary Care | Yes |  | Practice extract |
|  | - People Aged 65 and Over Who Received Both an Antiplatelet and an Anticoagulant in Quarter | Yes |  | There is sector confusion on this issue |

|  | MENTAL HEALTH STACK | Retained round 1 | Retained round 2 | Comment |
| --- | --- | --- | --- | --- |
|  | - Prescription of Selective Serotonin Re-uptake Inhibitor (SSRI) for the Management of People with Common Mental Disorders | Yes |  | How soon seen after new prescription |
|  | - Follow Up of Patients with Common Mental Disorders | Yes |  | Practice extract |
|  | - Mental Health Readmission % - 28 day acute inpatient readmission rate | Yes |  |  |
|  | - Prevalence of Common Mental Disorders in (i) Adults and (ii) Children | Yes |  | Practice extract |
|  | - Risk Assessment of People with Common Mental Disorders with a Focus on Depression | Yes |  | Practice extract |

|  | STAYING HEALTHY STACK – duplicates removed | Retained round 1 | Retained round 2 | Comment |
| --- | --- | --- | --- | --- |
|  | - Smoking Status Ever Recorded | Yes |  |  |
|  | - Management of Skin Infections in Primary Care | Yes |  |  |

**Health Quality and Safety Commission: Atlas of Variation**

https://public.tableau.com/profile/hqi2803#!/vizhome/PHOanalysis/PHOanalysis?publish=yes

|  | **ATLAS OF VARIATION** | **Retained round 1** | **Retained round 2** | **Comment** |
| --- | --- | --- | --- | --- |
|  | **Diabetes** |  |  |  |
|  | Prevalence of diabetes by district health board as per the Virtual Diabetes Register (VDR), percent | Yes |  |  |
|  | People with diabetes aged 25 and over regularly receiving metformin in a year, percent | Yes |  |  |
|  | People with diabetes regularly receiving insulin in a year, percent | Yes |  |  |
|  | People with diabetes aged 25 and over regularly receiving either metformin or insulin in a year, percent | Yes |  |  |
|  | People with diabetes regularly receiving ACEI or ARB in a year, percent | Yes |  |  |
|  | People with diabetes admitted one or more times to hospital with diabetic ketoacidosis, percent | No |  | Numbers too small |
|  | People with diabetes admitted to hospital with hypoglycaemia, percent | No |  | Numbers too small |
|  | People with diabetes having lower limb amputation, percent | No |  | Numbers too small |
|  | Proportion of medical-surgical bed days for people with diabetes compared to those without diabetes, percent | Yes |  |  |
|  | People with diabetes having regular HbA1c monitoring, percent | Yes |  | Ideally want level of HbA1c |
|  | People with diabetes having regular screening for renal disease (ACR), percent | Yes |  |  |
|  | People with diabetes having regular monitoring for renal disease (eGFR), percent | Yes |  |  |
|  | People with diabetes receiving all three laboratory tests | Yes |  |  |
|  | **Polypharmacy** |  |  |  |
|  | People aged 65 and over who received the ‘triple whammy’ | Yes |  |  |
|  | People aged 65 and over dispensed five or more unique long-term medications | Yes |  | Want just 1-2? |
|  | People aged 65 and over dispensed five, six or seven unique long-term medications | Yes |  | Need measure of quality |
|  | People aged 65 and over dispensed 8, 9 or 10 unique long-term medications | Yes |  |  |
|  | People aged 65 and over dispensed 11 or more unique long-term medications | Yes |  |  |
|  | People aged 65 and over who received an antipsychotic | Yes |  |  |
|  | People aged 65 and over who received a benzodiazepine or zopiclone | Yes |  |  |
|  | People aged 65 and over who received both a benzodiazepine or zopiclone and an antipsychotic in the same quarter | Yes |  | Numbers too small? |
|  | People who received both a benzodiazepine/zopiclone and a strong opioid following a public hospital event | Yes |  | Numbers too small? |
|  | **Asthma** |  |  |  |
|  | Children admitted to hospital one or more times in a year with a primary diagnosis of asthma or wheeze | Yes |  | Number of children immunised against flu |
|  | Adults aged 15 to 49 years admitted one or more times with a primary diagnosis of asthma | Yes |  |  |
|  | People with at least two admissions with a primary diagnosis of asthma or wheeze for children within 90 days of each other | Yes |  |  |
|  | People with at least two admissions with a primary diagnosis of asthma or wheeze in children within 91-365 days of each other | Yes |  |  |
|  | People not dispensed ICS regularly in the year after admission. | Yes |  |  |
|  | People (0 - 49) not given a funded influenza vaccine in the year after admission | Yes |  |  |
|  | People regularly dispensed SABA who were not dispensed preventer during the year | Yes |  |  |
|  | People regularly dispensed SABA and not regularly dispensed preventer during the year | Yes |  |  |
|  | **Gout** |  |  |  |
|  | The prevalence of identified gout in those enrolled with PHOs, by DHB (percent) | Yes | Yes |  |
|  | People with identified gout who received urate-lowering therapy regularly, by DHB (percent) | Yes | Yes |  |
|  | The dispensing of funded NSAIDs in those with identified gout, by DHB (percent) | Yes | Yes |  |
|  | The number of colchicine dispensings in people with identified gout not dispensed urate-lowering therapy, by DHB (percent) | Yes |  | Numbers too small? |
|  | The dispensing of funded NSAIDs in those with identified gout who were not dispensed urate-lowering therapy, by DHB (percent) | Yes |  |  |
|  | Serum urate testing in the six months following urate-lowering therapy dispensing, by DHB (percent) | Yes |  | Numbers too small? 1 year better |
|  | The number of admissions with a primary diagnosis of gout, by DHB (rate per 100,000) | Yes |  | Numbers too small? |
|  | The dispensing of funded NSAIDs in the rest of the New Zealand resident population, by DHB (percent) | Yes |  |  |
|  | **Opioid** |  |  |  |
|  | Crude rates per 1000 of ‘strong’ opioid dispensing by DHB | Yes | Yes | Use one of these |
|  | People dispensed a ‘strong’ opioid for 6 weeks or more, by DHB | Yes | Yes |  |
|  | Crude rates per 1000 of weak opioid dispensing by DHB | Yes |  |  |
|  | Crude rates per 1000 of tramadol dispensing | Yes | Yes |  |
|  | Crude rates per 1000 of codeine dispensing | Yes |  |  |
|  | Crude rates per 1000 of morphine dispensing by DHB | Yes |  |  |
|  | People dispensed morphine for 6 weeks or more, by DHB | Yes |  |  |
|  | Crude rates per 1000 of oxycodone dispensing by DHB | Yes |  |  |
|  | People dispensed oxycodone for 6 weeks or more, by DHB | Yes |  |  |
|  | People dispensed fentanyl, by DHB | Yes |  |  |
|  | People dispensed fentanyl for 6 or more weeks, by DHB | Yes |  |  |
|  | People dispensed a strong opioid who had a public hospital event in the 8 days prior to dispensing | Yes |  |  |
|  | **Antibiotic** |  |  | Consider pimafucort and similar |
|  | People dispensed one or more systemic antibiotics in a year, rate per 100 | Yes | Yes |  |
|  | All systemic antibiotic dispensings in a year, rate per 100 | Yes |  |  |
|  | People listed in ARC dispensed one or more systemic antibiotics in a year, rate per 100 | Yes |  |  |
|  | People dispensed a topical antibiotic in a year, rate per 100 | Yes | Yes |  |
|  | Seasonal variation in community systemic antibiotic dispensing, percent | Yes |  |  |
|  | People dispensed a broad-spectrum penicillin as a percent of all people dispensed a penicillin | Yes |  |  |
|  | People dispensed amoxicillin with clavulanic acid one or more times in a year, rate per 100 | Yes |  |  |
|  | People dispensed an antibiotic specifically indicated for urinary tract infections (UTIs), rate per 100 | Yes |  |  |
|  | People listed in ARC dispensed an antibiotic specifically indicated for UTIs, rate per 100 | Yes |  |  |
|  | Medical hospital admissions with an antibiotic dispensed within 30 days of discharge, rate per 100 | Yes |  |  |
|  | Surgical hospital admissions with an antibiotic dispensed within 30 days of discharge, rate per 100 | Yes |  |  |
